# Development of a highly sensitive liquid biopsy platform to detect clinically-relevant cancer mutations at low allele fractions in cell-free DNA

**DOI:** 10.1371/journal.pone.0194630

**Published:** 2018-03-16

**Authors:** Davina Gale, Andrew R. J. Lawson, Karen Howarth, Mikidache Madi, Bradley Durham, Sarah Smalley, John Calaway, Shannon Blais, Greg Jones, James Clark, Peter Dimitrov, Michelle Pugh, Samuel Woodhouse, Michael Epstein, Ana Fernandez-Gonzalez, Alexandra S. Whale, Jim F. Huggett, Carole A. Foy, Gerwyn M. Jones, Hadas Raveh-Amit, Karin Schmitt, Alison Devonshire, Emma Green, Tim Forshew, Vincent Plagnol, Nitzan Rosenfeld

**Affiliations:** 1 Inivata Ltd, Granta Park, Cambridge, United Kingdom; 2 Inivata Inc, Research Triangle Park, NC, United States of America; 3 LGC, Teddington, Middlesex, United Kingdom; 4 School of Biosciences & Medicine, Faculty of Health & Medical Science, University of Surrey, Guildford, United Kingdom; 5 Horizon Discovery, Waterbeach, Cambridge, United Kingdom; CNR, ITALY

## Abstract

**Introduction:**

Detection and monitoring of circulating tumor DNA (ctDNA) is rapidly becoming a diagnostic, prognostic and predictive tool in cancer patient care. A growing number of gene targets have been identified as diagnostic or actionable, requiring the development of reliable technology that provides analysis of multiple genes in parallel. We have developed the InVision™ liquid biopsy platform which utilizes enhanced TAm-Seq™ (eTAm-Seq™) technology, an amplicon-based next generation sequencing method for the identification of clinically-relevant somatic alterations at low frequency in ctDNA across a panel of 35 cancer-related genes.

**Materials and methods:**

We present analytical validation of the eTAm-Seq technology across two laboratories to determine the reproducibility of mutation identification. We assess the quantitative performance of eTAm-Seq technology for analysis of single nucleotide variants in clinically-relevant genes as compared to digital PCR (dPCR), using both established DNA standards and novel full-process control material.

**Results:**

The assay detected mutant alleles down to 0.02% AF, with high per-base specificity of 99.9997%. Across two laboratories, analysis of samples with optimal amount of DNA detected 94% mutations at 0.25%-0.33% allele fraction (AF), with 90% of mutations detected for samples with lower amounts of input DNA.

**Conclusions:**

These studies demonstrate that eTAm-Seq technology is a robust and reproducible technology for the identification and quantification of somatic mutations in circulating tumor DNA, and support its use in clinical applications for precision medicine.

## Introduction

Circulating cell-free DNA (cfDNA) from cancer cells, commonly referred to as circulating tumor DNA (ctDNA), is known to be present in the plasma of cancer patients. Since the first report of identical DNA mutations in plasma compared to a patient’s tumor, ctDNA has been investigated as a tool for cancer diagnosis, detection, prognostication, treatment selection and monitoring [1–3]. Over the past decade, increasing evidence demonstrates the utility of ctDNA as a ‘liquid biopsy’ to supplement conventional biopsies for molecular characterization and monitoring of solid cancers [4–7]. Circulating tumor DNA can be readily accessed from a non-invasive blood draw, allowing easier access to genomic information from a patient’s tumor or metastases as the cancer evolves, without the associated expense, complications or risk to patients during surgery or biopsy. Moreover, tissue testing may not be a viable option in many patients. In the Iressa Pan-Asia Study (IPASS), a phase III randomized study of gefitinib vs. carboplatin/paclitaxel in patients with pulmonary adenocarcinoma, *EGFR* mutation status could only be evaluated in 437/1038 (42%) patients that gave their consent for biomarker analyses [8]. The high failure rate may be due to a number of reasons, including insufficient biopsy material available, because the biopsy was of too poor quality for adequate analysis, or because surgery was not possible for medical reasons. In such cases, ctDNA can provide a valuable alternative for molecular stratification to select appropriate therapy. With the development of targeted therapies, the molecular profile of the cancer has been established to be informative to select therapies that are more likely to be effective in given patient groups. For example, tyrosine kinase inhibitors (TKIs), such as gefitinib and erlotinib, have been shown to be effective in non-small cell lung cancer (NSCLC) patients carrying activating *EGFR* exon 19 deletions or L858R mutations, and vemurafenib is known to be beneficial to patients with *BRAF* V600E mutations [9–11]. It has also been shown that it is possible to detect tumor evolution in plasma ctDNA [6, 12, 13]. *EGFR*-mutant NSCLC patients can now be tested and monitored to identify the emergence of newly arising *EGFR* T790M resistance mutations, and be effectively treated with osimertinib, a third generation TKI [14–15].

Studies have shown that ctDNA levels often correlate with tumor burden, and provide an earlier and potentially more reliable measure of treatment response than other clinical biomarkers, such as CA-15-3 in metastatic breast cancer, and CA-125 in advanced high-grade serous ovarian cancer [5, 7]. Recent exciting developments have shown that it is possible to use ctDNA as a tool to assess minimal residual disease [16], and be used to identify mutant DNA in early stage cancer, although this is much more challenging given the lower number of mutant molecules present in the bloodstream [17,18]. With such diversity in potential clinical applications, it is important to use a ctDNA assay that has high sensitivity and specificity, and can interrogate multiple mutations in parallel to detect, track and monitor clinically-relevant genomic changes as the cancer evolves. Several techniques are available for the analysis of ctDNA. Many of the earlier studies focused on analyzing single mutated regions. Digital PCR (dPCR) and BEAMing have both been established as sensitive techniques for the detection and quantification of specific ‘hotspot’ mutant alleles [19, 4]. The cobas *EGFR* Mutation Test v2 is a real-time PCR test for the qualitative detection of *EGFR* exon 19 deletions, L858R and T790M mutations, and is used to determine which NSCLC patients are eligible for treatment with erlotinib or osimertinib. The test has gained FDA-approval for testing on both plasma and tissue, making it the first companion diagnostic that allows the use of ctDNA analysis to guide treatment [20]. The FDA-approved assays, however, are less sensitive than digital PCR, with a limit of detection (LOD) at >25 copies/mL of plasma [21]. Analysis of single genomic loci is restricted to a limited number of pre-defined hotspots. To analyse multiple mutations, cfDNA must first be sub-divided for each assay, reducing the sensitivity of the test and introducing potential sampling bias for detection of low frequency alleles.

The development of next generation sequencing (NGS) has allowed for a broader application of ctDNA analysis. In 2012, Forshew et al. developed TAm-Seq technology, or tagged-amplicon deep sequencing which, for the first time, enabled interrogation of 6 genes across a large genomic region spanning 5995 bases to detect low frequency mutations in cell-free DNA [22]. The assay was evaluated in plasma from patients with high-grade serous ovarian cancer, and shown to have 97% sensitivity and specificity for detection of mutations at 2% allele fraction (AF), and was able to identify mutations down to 0.14% AF. Analysis of clinical samples showed that it is possible to use TAm-Seq technology to assay multiple mutations in parallel to monitor tumor dynamics, identify *de novo* mutations direct from patient cfDNA, and identify the origin of metastatic relapse. Since this time, other NGS technologies have been implemented for analysis of ctDNA, including the use of hybrid capture and the introduction of panel assays that use molecular barcodes to enable error suppression [6, 23, 24]. The ideal ctDNA assay needs to have high sensitivity and specificity, have good turnaround times and target clinically-relevant and clinically actionable genes. This will enable oncologists to make clear treatment decisions based on molecular profiling information, according to cancer care guidelines and used in conjunction with other clinical observations.

Here we describe the development of the InVision liquid biopsy platform which utilizes enhanced TAm-Seq (eTAm-Seq^TM^) technology for the identification of low frequency mutations in ctDNA. The assay has been expanded to target hotspots and entire coding regions from 35 cancer-related genes, utilizing a primer design strategy that allows for amplification of highly fragmented DNA, typical of ctDNA. The calling algorithm has been revised, and in addition to improved detection of single nucleotide variants (SNVs) and short insertions/deletions (indels), it also identifies copy number variants (CNVs). The library preparation process has been adapted to remove the use of microfluidics and to reduce the background error rate. We present analytical validation of the InVision liquid biopsy platform across two laboratories to demonstrate its reproducibility and to support the use of this platform in clinical applications. We compare the performance of eTAm-Seq technology and digital PCR by analysis of sheared cell-line reference standard DNA and novel full-process control material developed by LGC and Horizon Discovery.

## Materials and methods

### Analytical validation of eTAm-Seq technology

To assess the performance of the eTAm-Seq technology, analytical validation studies were performed in two laboratories within the scope of CLIA (Laboratory 2) and ISO 15189:2012 quality standards (Laboratory 1). Next-generation sequencing libraries were prepared using eTAm-Seq technology, analysing sheared reference standard DNA and cfDNA extracted from control plasma from presumed healthy controls. Healthy control samples used in this study were obtained on a commercial basis, from BioreclamationIVT (US) and Seralab (UK). Cell-free DNA (cfDNA) was extracted from 5 mL plasma using a QIAamp Circulating Nucleic Acid kit (Qiagen) as previously described [22], incorporating an internal control to monitor extraction efficiency. cfDNA and the internal control were both quantified by dPCR, using either the Fluidigm Biomark or Biorad QX200, with a 108 bp assay targeting a region of ribonuclease P/MRP subunit p30 (*RPP30*) gene (Forward = 5’-GGAGGTGGAGGAGGAGGATA-3’; Reverse = 5’-ACGGAATACAGAACCCATGACT-3’; Probe = 5’-FAM/AGCCTTGAG/ZEN/ AGACGAGAACCTGT/IABkF Q-3’) and an assay targeting the internal control, as previously described [22]. Yields were expressed as amplifiable copies (AC) per 10 mL blood.

#### Preparation of sheared cell-line reference standard DNA for analytical validation studies

Horizon Tru-Q 6 (2.5% Tier) and Tru-Q 7 (1.3% Tier) reference cell-line DNA samples (Horizon Discovery), carrying cancer-related mutations at known allele fractions, were sheared to ~200 bp by acoustic shearing (Covaris) to mimic fragmented cfDNA. Tru-Q 6 contains a mix of 20 isogenic genetically-engineered cell lines with known engineered and endogenous mutations predominantly at ~2%-2.5% AF (range: <2%-30% AF), and Tru-Q 7 contains a mix of 40 cell lines with known mutations predominantly at ~1%-1.3% AF (range: <1%-30% AF). Dilutions were prepared using sheared Horizon Tru-Q 0 wild-type DNA as diluent.

#### InVision liquid biopsy tumor profiling panel

**Fig 1:**
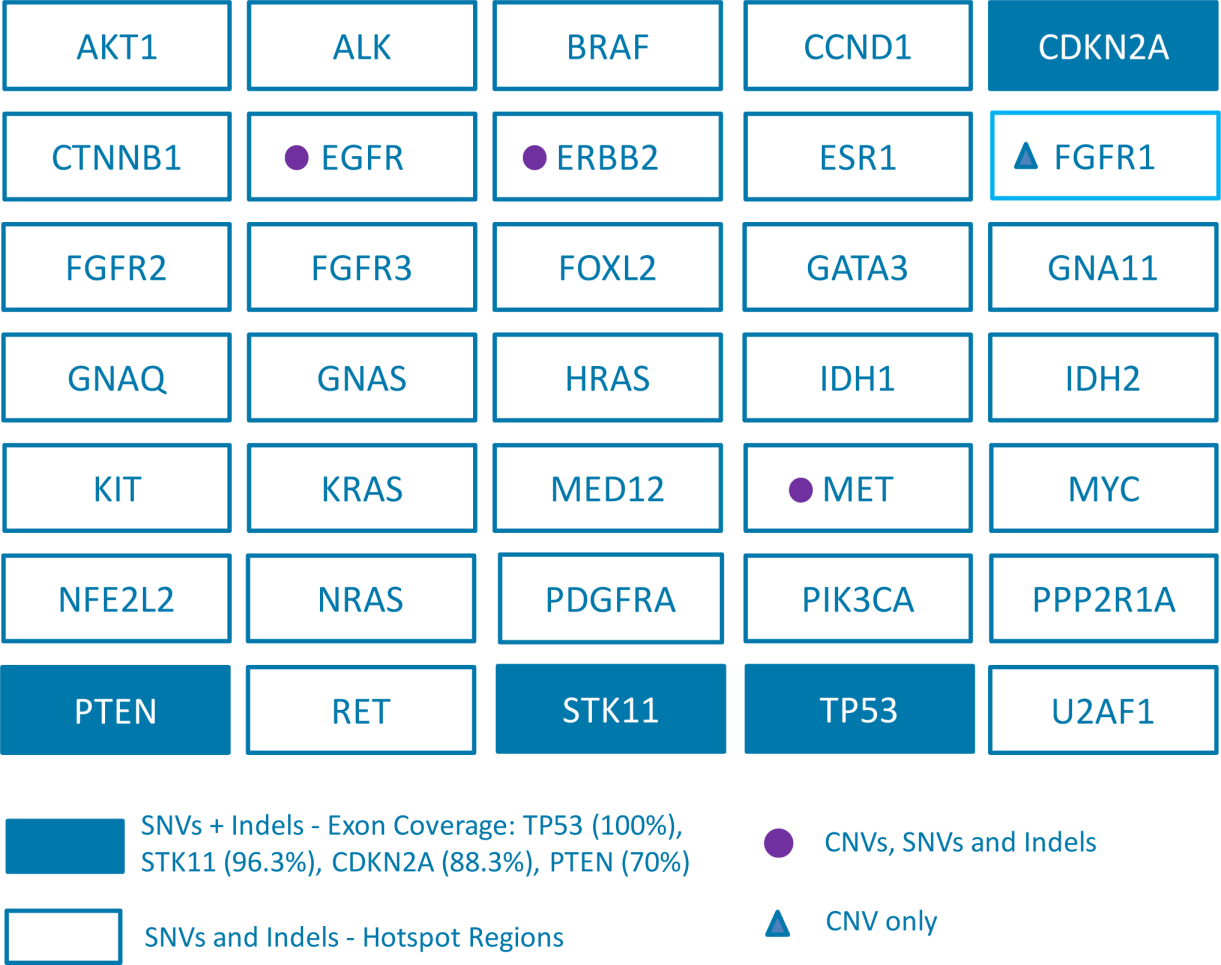
InVision liquid biopsy tumor profiling panel. The coverage per gene is indicated, including hotspots, comprehensive or full coverage of coding regions (70%–100% tiling coverage) and CNVs. SNVs = Single Nucleotide Variants; Indels = short insertions or deletions; CNVs = Copy Number Variants.

The InVision liquid biopsy platform utilizes an enhanced version of TAm-Seq technology to identify and quantify low frequency tumor-derived SNVs and indels in cfDNA. The technology is also able to identify CNVs in *EGFR*, *ERBB2* (also known as *HER2*), *FGFR1* and *MET* [25]. Full analytical validation of CNVs is not included in this study. The assay targets 35 cancer-related genes spanning 10.61kb, using primers designed to hotspots and entire coding regions of interest. Covered regions were chosen to maximise the mutation yield for common cancer types primarily NSCLC, focusing on clinically actionable mutations. We therefore included oncogenes *EGFR*, *BRAF*, *KRAS*, *ERBB2*, *MET* (exon 14), *U2AF1*, *CTNNB1*, *EGFR*/*MET* amplifications as well as tumour suppressor genes *TP53*, *STK11*, *PTEN*. We further included key regions of *ESR1*/*GATA3*, as well as *ERBB2*/*FGFR1* amplifications, and the most common mutation hotspots in common carcinomas as defined by COSMIC frequencies. The panel was designed optimizing primers for amplification of fragmented DNA with amplicon sizes ranging from 72bp-154bp. The primers were selected based on factors including GC content, similar Tm (target 60°C), avoidance of primer dimer, avoidance of off-target products and avoidance of SNPs. Fig 1 shows an overview of the InVision liquid biopsy tumor profiling panel, and S1 Table provides detail of the exonic regions covered.

#### Library preparation using eTAm-Seq technology

eTAm-Seq technology is based on methods previously described [22, 25,26], with an optimized assay workflow utilizing multiplex PCR to enable high-throughput library preparation without the use of microfluidics. Next generation sequencing libraries were prepared using a two-step multiplex PCR amplification process incorporating replicate and patient-specific barcodes and Illumina sequencing adaptors. Different input amounts of DNA were used to assess the performance of the assay, using either 2,000 AC (low), 8,000 AC (medium) or 16,000 AC (high) input (~6.6ng to 53ng of amplifiable DNA). All regions were analysed multiple times using a fixed DNA input range for all samples to enable error correction [26]. As each sample is analysed multiple times, false positive and true positive calls can be readily identified, providing a robust analytical pipeline [22, 26]. After target enrichment, amplified regions were purified using SPRISelect beads (Beckman Coulter) following the manufacturer’s protocol. Samples were quantified using the LabChip GX touch and DNA high sensitivity assay. Quantified samples were then pooled to generate a normalized library of 12 nM. This library was quantified using the Kapa Library Quantification Kit, and 1.8 pM libraries analysed on an Illumina NextSeq 500 (300 cycle PE) with 5% PhiX to monitor sequencing performance.

#### Data analysis

Sequencing files were analysed using the Inivata Somatic Mutation Analysis (ISoMA) pipeline to identify SNVs, CNVs and indels. A minimum Phred quality score of 30 for each base was required for inclusion in the analytics. The pipeline clipped primers and merged paired-end reads into synthetic reads (using Flash v1.2.11). A minimum Phred quality score of 2 was assigned to discordant positions at the merging step. Default settings were used for Flash and a Phred quality score of 2 was assigned to mismatched base pairs. These synthetic reads were subsequently aligned to the reference genome using BWA (v0.7.12). Samples passing sequencing QC were kept for further analysis.

To enable variant calling, the background noise for each potential SNV was compared to the variability observed from a set of control samples [22]. The same statistical principle was used for indels using samples from the same batch of samples in order to enable appropriate background calibration. In addition, each run was assessed using positive and negative controls. Common single nucleotide polymorphisms (SNPs) were used to identify potential cross sample contamination, as well as rule out potential swaps for longitudinal studies involving multiple samples from the same patient. The final determination of a call integrated the data across replicates for the sample within a maximum likelihood framework. Variants were annotated using the variant effect predictor [27] based on the canonical transcript for each gene. SNVs and indels that resulted in coding and splice-site mutations were reported. For CNVs, a normalized measure of read depth that corrects for sample and amplicon effects was used to infer the number of DNA copies. A mutation calling report was generated providing a comprehensive summary of somatic alterations identified.

### Comparison of performance of eTAm-Seq technology and digital PCR by analysis of novel full-process control material

#### Preparation of pooled plasma

Sixteen human plasma samples of ~20 mL each from 6 male and 10 female donors were obtained from Seralab (UK). All plasma samples had undergone a second centrifugation step of 1,000 x *g* for 10 minutes at 4°C, following initial centrifugation from whole blood. Samples were stored at -80°C upon receipt. Samples were pooled and homogenized using a roller mixer for 30 minutes at 4°C followed by preparation of 5.0 mL aliquots which were frozen at -80°C.

#### cfDNA reference standards

Multiplex I cfDNA Reference Standards (Horizon Discovery) were generated from genomic DNA isolated from isogenic cell-lines, and fragmented to ~160 bp by acoustic shearing (Covaris). The standards, containing 8 known mutations in *EGFR* (L858R, Δ746–750, T790M, V769-D770insASV), *KRAS* (G12D), *NRAS* (A59T, Q61K) and *PIK3CA* (E545K), were diluted to 8 ng/μL for spiking into plasma.

#### cfDNA extraction

Following thawing of plasma aliquots, 50 μL (400 ng) of Multiplex I cfDNA Reference Standard containing target mutations at ~5%, ~1%, ~0.1% AF or 100% wild-type DNA was added to 5 mL pooled plasma and mixed by vortexing for 10 seconds. DNA was extracted from plasma samples using the QIAamp Circulating Nucleic Acid Kit (Qiagen) and eluted in 50 μL AVE buffer. Replicate extractions (n = 6) were performed for all four levels of Reference Standard (5%, 1%, 0.1% and 100% wild-type) and plasma only controls over 3 days (2 extractions per day). Extracts were divided into two aliquots (25 μL) and frozen, with one aliquot analysed by the eTAm-Seq technology and one aliquot analysed by digital PCR.

#### Mutational analysis

Samples were analysed using the eTAm-Seq technology in Laboratory 1 using an average of 12,450 AC per reaction. Digital PCR analysis was performed using a QX200 droplet dPCR system (Bio-Rad) with a C1000 Touch Thermal Cycler (Bio-Rad) at LGC. *KRAS* G12/WT and *EGFR* L858R/WT mutations were assessed using PrimePCR assays (Bio-Rad) and custom designed assays were used targeting *NRAS* A59T/WT and *PI3KCA* E545K/WT (S2A–S2C Table). Primers and BHQplus probes for custom assays were supplied by BioSearch and diluted in 1 x TE pH 8.0 (Sigma). Reactions (20 μL) were prepared (with 10% excess) and contained ddPCR Supermix for Probes with no dUTP (Bio-Rad), 20x primer/probe mix, 4 μL cfDNA extract (n = 1 per target mutation) with the remaining volume nuclease-free water (Ambion). Non-spiked Multiplex I cfDNA Reference Standards (32 ng/reaction) were analysed alongside the spiked extracts as controls (n = 3). Data was analysed using QuantaLife (Bio-Rad, version 1.6.6.0320) with classification of single positive, double positive and negative droplets as shown in S1A–S1E Fig. Copy number concentration was calculated based on a partition volume of 0.85 nL.

#### Calculation of LOD for dPCR assays

The LOD of dPCR assays were calculated using the approach described in Whale et al. based on modelling two binomial distributions, combining a 5% probability of a false positive (α = 0.05) with a 5% probability of a false negative (β = 0.05) [28]. The false positive rate (FPR) for each assay was calculated from analysis of the 100% WT Multiplex I cfDNA Reference Standard (n = 6 reactions, 80 ng per reaction). The ‘critical level’ is the 95th percentile for a binomial distribution with n trials and probability given by the false positive rate per droplet (λ), where n is the mean number of droplets from the data. The LOD expressed as mutant copies per reaction is given by n ×–ln(1-p) where p is the probability of success for the binomial distribution with n trials and where the 5th percentile equals the critical level. The LOD expressed as AF% is the previous value relative to the total number of target copies (i.e. n times the mean concentration per droplet (λ) of the wild-type target, plus the mutant value) (S3 Table).

## Results

### Analytical validation of the eTAm-Seq technology

Analytical validation studies were performed to assess sensitivity of the eTAm-Seq technology for detection of SNVs and indels. Horizon Tru-Q 6 Tier 2.5% and Tru-Q 7 Tier 1.3% cell-line reference standard DNA, carrying mutations at known AF were sheared to ~200bp to approximate cfDNA. There are 21 mutations present in Tru-Q6, and 38 mutations in Tru-Q7 targeted by the InVision liquid biopsy tumor profiling panel. Dilutions were prepared using Horizon Tru-Q 0 wild-type DNA as diluent. Data previously published showed that concentrations of cfDNA in plasma of cancer patients was >1.65 ng/mL in 99% of patients, >6.6 ng/mL in 80% of patients, and >13.2 ng/mL in nearly 50% of patients [29]. This is consistent with cfDNA amounts in 10 mL blood samples (approximately 4~4.5 mL of plasma) from NSCLC patients which were previously shown to contain >2,000 AC (~6.6 ng) in >95% of samples, >8,000 AC (~26.4 ng) in >69% of samples and >16,000 AC (~52.8 ng) in >40% of samples [25]. Dilutions were therefore performed to prepare low (2,000 AC), medium (8,000 AC) and high (16,000 AC) input amounts. Limit of Detection (LOD), inter-operator and inter-laboratory variability were assessed by performing the assay across two laboratories by 6 operators on different days and sequenced on different NGS runs. Each operator independently performed the entire process, and one of the operators performed the assay at each of the two laboratories. Multiple assays were performed in each laboratory at different dilution levels, as shown in Table 1.

**Table 1:** Details of analytical validation experiments performed to assess sensitivity of the eTAm-Seq technology, including range of input DNA (AC), AF (%), number of sample repeats per operator, and number of operators per laboratory.

Input DNA (AC)	AF (%)*	Number of repeats/operator	Number of operators/laboratory
16,000	0.25%-0.33%	4	3
8,000	1%-1.3%	3	3
2,000	2%-2.5%	7	3
8,000	0.5%-0.65%	3	3
8,000	0.25%-0.33%	3	3
8,000	0.13%-0.16%	3	3
8,000	0.06%-0.08%	3	3

* AF shows indicative ranges for Tru-Q reference material, full list of values presented in S4 Table.

**Fig 2:**
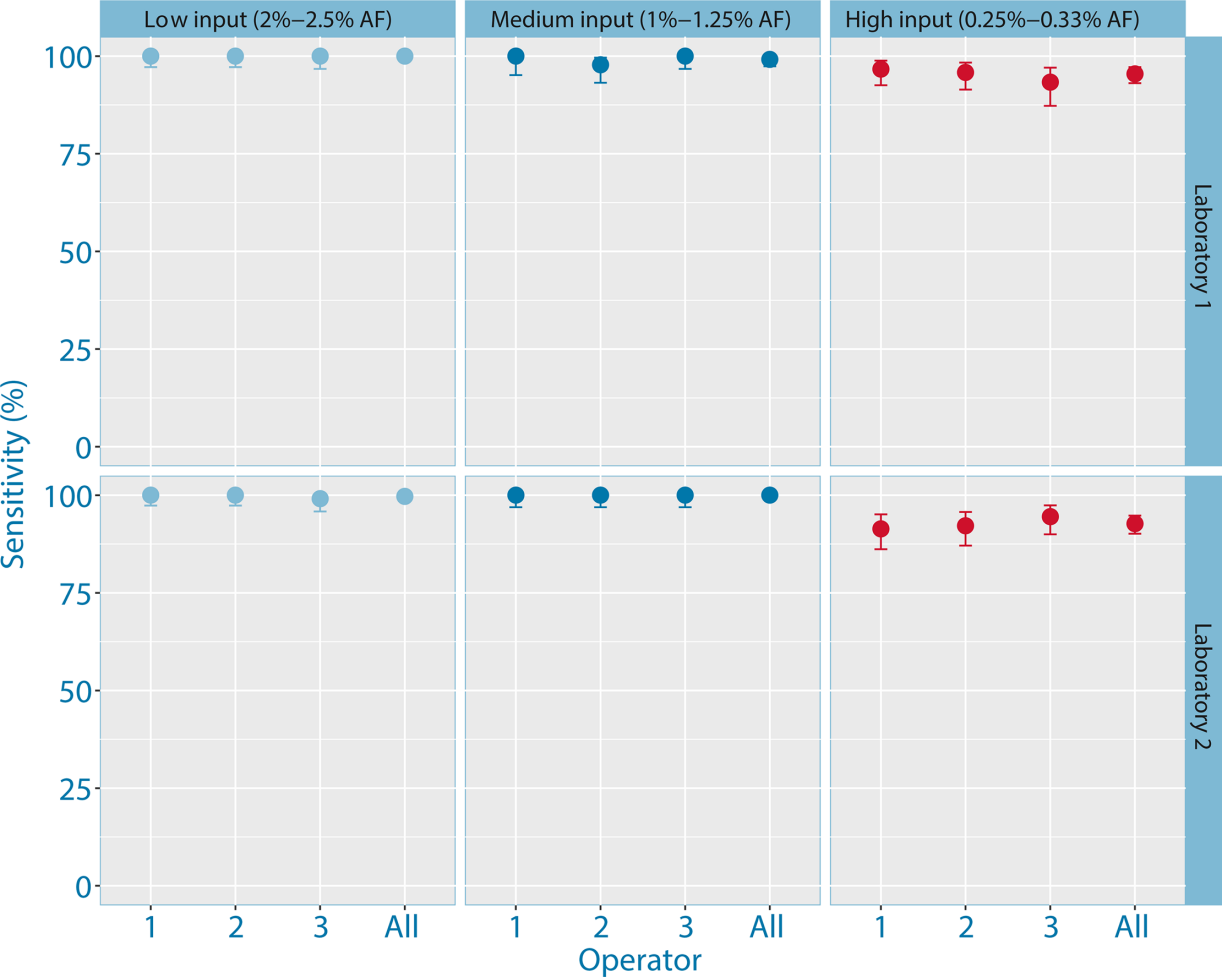
Plot showing sensitivity and inter-operator variability of eTAm-Seq technology using low, medium and high input DNA. Experiments were performed in two laboratories (Laboratory 1 –upper; Laboratory 2 –lower) by different operators, performed on separate days and different NGS runs.

Analysis using eTAm-Seq technology showed that the assay had high sensitivity (Fig 2). In Laboratory 1, sensitivity was 100% (90% confidence interval (CI): 99.01%-100%) in low input samples at 2%-2.5% AF, 99.17% (90% CI: 97.40%-99.85%) in medium input samples at 1%-1.3% AF, and 95.45% (90% CI: 93.09%-97.18%) in high input samples at 0.25%-0.33% AF. Comparable results were seen in the second laboratory, with a sensitivity of 99.7% (90% CI: 98.6%-99.98%) in low input samples, 100% (90% CI: 98.97%-100%) in medium input samples, and 92.71% (90% CI: 90.14%-94.77%) in high input samples.

To further assess the limit of detection (LOD) using medium input of 8000 amplifiable copies of DNA, a dilution series was created by spiking sheared Horizon Tru-Q 7 into Tru-Q 0 reference standard to approximate an AF range of 0.06%-1.25% AF. In Laboratory 1, 99.17% mutations were detected at 1%-1.3% AF, 99.63% at 0.5%-0.65% AF, 89.17% at 0.25%-0.33% AF, 69.26% at 0.13%-0.16% AF and 37.41% at 0.06%-0.08% AF. Comparable results were seen in Laboratory 2 (Table 2, S2 Fig, S4 Table, S5 Table) and for all samples (S6 Table).

**Table 2:** Sensitivity of the eTAm-Seq technology with 8000 amplifiable copies of DNA input per sample.

	Laboratory 1			Laboratory 2		
AF (%)*	Sensitivity (%)	90% CI (Lower)	90% CI (Upper)	Sensitivity (%)	90% CI (Lower)	90% CI (Upper)
**1%-1.3%**	99.17	97.40	99.85	100.00	98.96	100.00
**0.5%-0.65%**	99.63	98.26	99.98	97.66	95.43	98.97
**0.25%-0.33%**	89.17	85.29	92.30	90.28	86.91	93.00
**0.13%-0.16%**	69.26	64.31	73.89	67.71	62.88	72.26
**0.06%-0.08%**	37.41	32.50	42.52	30.86	26.10	35.95

* AF shows indicative ranges for Tru-Q reference material, full list of values presented in S4, S5 and S6 Tables.

Across the two laboratories using 8000 amplifiable copies of input DNA, 98.65% of mutations were detected at 0.5%-0.65% AF. For the 0.25%-0.33% dilution range, the sensitivity was 89.73%. The lowest frequency mutation identified was an *EGFR* indel (ΔE746-A750) detected at 0.02% AF.

### Assessment of quantitative performance of eTAm-Seq technology by comparison with digital PCR analysis of reference cell-line DNA carrying mutations at known allele fraction

**Fig 3:**
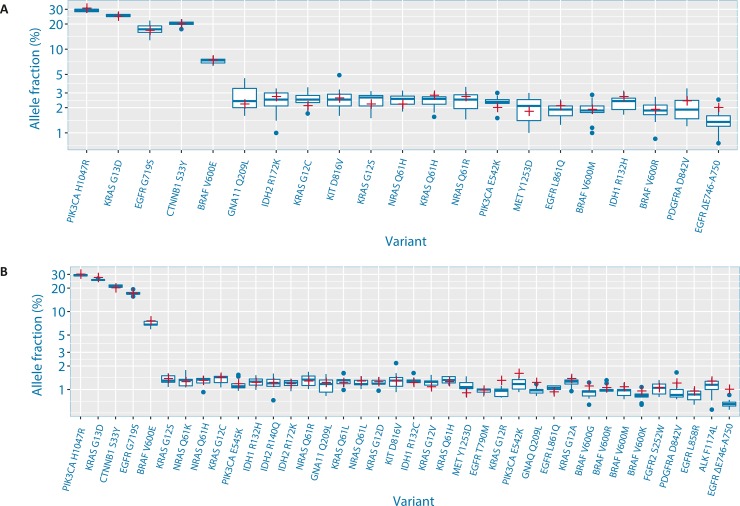
Plot showing allele fractions determined by analysis with eTAm-Seq technology (blue boxplot) and digital PCR (red cross) for analysis of mutations present in both the InVision liquid biopsy tumor profiling panel and (A) Tru-Q 6 and (B) Tru-Q 7.

In order to determine the quantitative performance of the eTAm-Seq technology, data was compared with allele fractions generated by digital PCR analysis of Horizon Tru-Q 6 and Tru-Q 7, supplied by the manufacturer. As can be seen in Fig 3A and Fig 3B, there is good concordance between AFs determined by the eTAm-Seq technology and digital PCR analysis of 21 mutations present in both the InVision liquid biopsy tumor profiling panel and Tru-Q 6, and analysis of 38 common mutations in Tru-Q 7. This demonstrates the quantitative accuracy of eTAm-Seq technology for reliable detection of mutations at low allele frequency.

### Assessment of specificity of the eTAm-Seq technology by analysis of plasma from presumed healthy donors

Tru-Q6 or Tru-Q7 reference DNA contains additional mutations outside of the validated mutations listed in these cell-line mixes, and is therefore not suitable for assessing specificity. Plasma samples from 79 presumed healthy donors were therefore analysed using eTAm-Seq technology to assess specificity. This analysis identified five low frequency coding mutations, all at ≤0.5% AF: three located in *TP53* [L308L at 0.19% AF (Laboratory 1); Y220C at 0.5% AF and P27L at 0.5% AF (Laboratory 2)] and two in *GATA3* [T323T at 0.1% AF and T419T at 0.317% AF (Laboratory 2)]. Sufficient material was available to enable re-extraction of plasma cfDNA from the same blood draw in four out of five cases (all but *GATA3* T419T). Analysis by eTAm-Seq technology was repeated for these 4 samples. Re-analysis confirmed the initial call for three of the four samples, failing only to detect the *TP53* L308L change originally identified at 0.19% AF. This resulted in two potential false positives, one is unconfirmed, and the other may be a false-negative of the replicate assay at ≤0.19% AF. The identification of 2 potential false positives in 79 healthy samples amounts to a per-base specificity of at least 99.9997% (95% confidence interval, 99.9989% to 99.99996% per-base specificity).

### Analysis of novel full-process control material using eTAm-Seq technology and digital PCR

**Fig 4:**
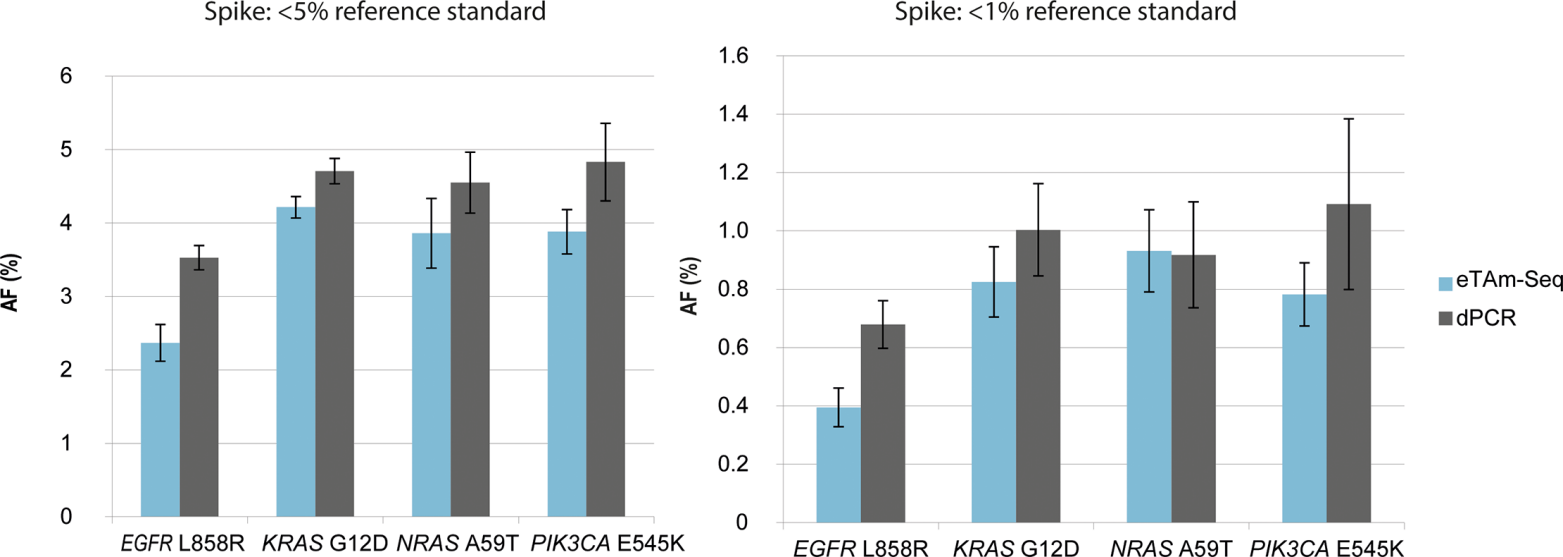
Quantitative agreement of 5% AF and 1% AF reference standard spiked into plasma, and measured by eTAm-Seq technology and dPCR. Mean mutant AF (%) ± SD are displayed for each technology (n = 5* (5% AF standard); n = 6 (1% AF standard)). By spiking into plasma containing background wild-type DNA, the resulting mix was confirmed to contain lower AFs than the original reference standards (original mutant AF values 5% standard: 5% (*EGFR*); 6.3% (*KRAS*, *NRAS*, *PIK3CA*); 1% standard: 1% (*EGFR*), 1.3% (*KRAS*, *NRAS*, *PIK3CA*). (*1 data point omitted due to anomalous extraction efficiency).

To explore the performance of a novel full-process control with spiked DNA reference standards and assess the ability of the eTAm-Seq technology to identify low frequency mutations, 5 mL aliquots of pooled plasma from 6 male and 10 female presumed healthy donors were spiked with 400ng Multiplex I cfDNA Reference Standard. This reference standard, acoustically sheared to 160bp to mimic cfDNA, is derived from well-characterized isogenic cell-lines and contains 8 target mutations at ~5%, ~1% or ~0.1% AF. 100% wild-type DNA from non-modified cell-lines containing 100% wild-type DNA was used as a control. By spiking into plasma containing background DNA, the resulting mix would be expected to contain lower AFs than the original standards. For each of the four levels, replicate cfDNA extractions (n = 6) were performed over 3 days, together with replicate plasma-only controls. The cfDNA was sub-divided into two for analysis by both eTAm-Seq technology (Laboratory 1) and dPCR (LGC). dPCR analysis was performed targeting hotspot mutations in *EGFR* L858R, *KRAS* G12D, *NRAS* A59T and *PIK3CA* E545K. The observed extraction efficiency was highly reproducible between replicates, with ~50% recovery of the spike-in (S3 Fig). More variability was observed in the 0.1% AF-spiked sample, likely due to sampling noise when quantifying small numbers of mutant molecules. The extraction efficiency was slightly lower than has previously been reported (60%-80% recovery) for measurement of a spike-in control [30]. Quantification of the <5% AF and <1% AF samples using the eTAm-Seq technology and dPCR spiked plasma showed good concordance (Fig 4). The small deviation in AF observed in the contrived control samples may be related to differences in DNA fragment sizes between the sheared mutant DNA and the wild-type donor plasma, which may differentially affect the results of the two methods. Both methods showed good precision with low %CV for all 4 mutations in analysis of plasma spiked with 5% and 1% AF reference standard (S4 Fig).

100% of mutations known to be present in the <5% and <1% AF pool were detected by both eTAm-Seq technology and dPCR (S5 Fig, S7 Table). An additional 5 coding mutations in *BRAF* V600E (20.07% AF), *CTNNB1* S33Y (14.72% AF), *PIK3CA* H1047R (13.98% AF), *STK11* Q123Q (13.37% AF) and *EGFR* G719S (13.31% AF) were detected using the eTAm-Seq technology. These mutations were all confirmed to be present by exome sequence analysis of the original isogenic cell-lines that the reference standards were derived from. In the <0.1% spiked sample, across the 4 overlapping mutations analysed by both methods in the 6 replicate extractions, amplicon sequencing detected 11/24 mutations whilst dPCR detected 16/24. Overall, 25/48 (53%) mutations in the <0.1% AF sample were detected using eTAm-Seq technology, as expected given the limit of detection for the assay and stochastic sampling effects. Two potential false positives were identified: *TP53* F113V (GRCh38 chr17:7676032 A>C) at 0.15% AF and *GNA11* R214M (chr19:3118959 G>T) at 0.1% AF. The *GNA11* mutation was possibly caused by 8-oxoguanine (8-oxoG) lesions created during the shearing process used to create the original reference standard DNA. Many of the samples with spiked fragmented DNA had high background at this position and at other G bases, whilst non-sheared plasma did not show an aberrant base both in this run and in previous experiments. The *TP53* mutation was observed significantly above normal background, and may be a false positive or a true low frequency variant.

## Discussion

It has long been known that genomic alterations in cancer can be detected in the plasma of cancer patients in the form of circulating tumor DNA. Increasing evidence indicates clinical utility of ctDNA as a diagnostic, prognostic and predictive tool with potential application throughout the continuum of cancer care. FDA approval of the first companion diagnostic permitting ctDNA-based mutation detection, and the emergence of several ctDNA-guided clinical trials [31–33] signals growing acceptance of its utility. ctDNA analysis offers important advantages over profiling single biopsies taken during invasive surgery, often many months or years before clinical progression. ctDNA enables repeat sampling and molecular assessment of tumor evolution during patient treatment, which may help guide subsequent therapy [5, 15]. Advances in NGS have shown it is possible to monitor tumor dynamics and assess evolution in plasma by analysis of multiple mutations in parallel across serially-collected samples, rather than focusing on single hotspot mutations. Digital PCR analysis of multiple mutations is possible to a limited degree but requires sub-dividing DNA into different assays. When large amounts of DNA are available, this can be achieved but where DNA is limited, such as in the analysis of cfDNA, this results in sampling noise and loss of sensitivity as rare mutant molecules are missed. NGS analysis, with a sensitive and appropriately validated platform, circumvents these issues, providing substantially more information on somatic alterations present in the bloodstream, which can be used to guide subsequent cancer therapy.

Currently, there is a limited but growing number of clinically actionable gene targets. The hope is that future advances will result in the development of new immunotherapies and targeted treatments effective against additional somatic alterations known to be present. One important factor in the development of a clinically useful ctDNA assay is to strike the right balance in the size of the genomic region analysed to enable optimal test sensitivity and specificity. By increasing the size of the genomic region covered, the correction for false positives needs to be more stringent. Hybrid capture-based enrichment methods have enabled analysis of focused genomic regions up to whole exomes [6, 23, 24]. However, analysis of larger regions either requires expensive high depth sequencing to identify low frequency mutations, or a compromise on depth and associated reduction in sensitivity. Hybrid capture can be used to target more focused regions but this leads to a high proportion of off-target sequencing reads. Like the sampling noise challenge for dPCR described above, a key limit for all NGS methods developed for cfDNA analysis is the fraction of DNA molecules successfully analysed. Through PCR enrichment, with suitably short amplicons, amplicon-based sequencing can achieve sensitivity comparable to dPCR by amplifying and thereby sampling the majority of cfDNA molecules accessible to PCR amplification. Since samples need not be split into multiple assays, the effective sensitivity of amplicon-based sequencing may even exceed that of dPCR [20, 34, 35]. Hybrid capture library preparation methods are not restricted to amplifying regions containing both priming sites. However, they require considerable pre-processing prior to enrichment or PCR-based amplification and therefore may lose a significant proportion of molecules during library preparation stages, particularly during adaptor ligation [36]. This is important for analysis of ctDNA given the low frequency of tumor-derived DNA molecules present in patient plasma, particularly in earlier stage cancer.

Here, we have described the InVision liquid biopsy platform which utilizes enhanced TAm-Seq technology for the identification of low frequency mutations in cell-free DNA. This amplicon-based method has been carefully optimized for efficient amplification from limited amounts of fragmented plasma DNA. The focused gene panel targets 35 clinically actionable and clinically-relevant genes, providing coverage of critical regions in 31 genes and near complete coverage of 4 genes of clinical significance. Analytical validation of the assay demonstrates high sensitivity and specificity for detection of low frequency mutations with 94.08% of mutations detected at 0.25% - 0.33% allele fraction (AF) with optimal DNA input, with a per-base specificity of 99.9997%. Validation across two laboratories demonstrates its reproducibility and supports its use in clinical applications. In addition, the assay is highly quantitative, demonstrating excellent concordance with digital PCR analysis of commercial cell-line reference standard DNA, and novel full-process control material developed by LGC and Horizon Discovery, that carry cancer-related mutations at known allele fractions.

Using this assay, mutant alleles were detected down to 0.02% AF, with >30% sensitivity for detection at 0.06% AF (1 mutant DNA copy in 1600 molecules). This study identifies two challenges when assessing assay specificity using either individual donors or acoustically-sheared commercial reference standards for analysis of low frequency mutations in ctDNA using an ultra-sensitive test. During analysis, five mutations at ≤0.5% AF were identified in presumed healthy donors, yet when 4 samples with sufficient material were re-analysed, the same mutations were repeatedly identified in 3, indicating these were true positives; the 4^th^ change was originally detected at a low allele fraction of 0.19% so possibly missed on repeat due to its low allele fraction. Somatic mutations have previously been detected in presumed healthy individuals, and may represent pre-malignant mutations that accumulate prior to cancer or during the aging process [37], or changes that have arisen during clonal hematopoiesis [37, 38] or could originate from undetected tumors. More studies are needed using orthogonal assays with similarly high sensitivity to determine if changes are truly present. Using the commercial reference standard, a *GNA11* R214M G>T mutation was identified along with a signature of high background G>T/C>A errors at other bases. This is consistent with 8-oxoguanine (8-oxoG) lesions created during the acoustic shearing process or potentially evolution and heterogeneity of the cell lines. The phenomenon of G>T/C>A transversion artifacts was first identified by Costello et al. [39, 40], and highlights the potential risk of using acoustically-sheared DNA to validate specificity of sensitive ctDNA NGS-based assays capable of detecting low frequency sequence aberrations. One solution may be to limit the use of sheared material to the assessment of assay sensitivity, since this material performed well at the loci that were defined and tested for this purpose, and use donor (healthy volunteer) DNA for broader specificity assessment (given the caveats previously mentioned and repeat or orthogonal analysis for confirmation). Alternatively, different mechanisms could be investigated to fragment commercial reference standard DNA, such as enzymatic fragmentation, which may potentially introduce less DNA damage.

Given the restrictive requirement to immediately process EDTA-collected blood to plasma to prevent leukocyte lysis, it is important to validate the eTAm-Seq technology using blood collected into Streck Cell-free DNA BCT tubes. These tubes contain a proprietary cell preservative which stabilizes nucleated blood cells preventing contamination with background wild-type DNA. Analysis of the eTAm-Seq technology in EDTA and Streck tubes collected at the same time from patients has previously been presented [40], and showed high technical reproducibility between two independently processed blood tube types, indicating use of either tube type is suitable for clinical blood collection using this technology. The Streck Cell-free DNA BCT tubes provide a robust alternative to enable delayed and centralized processing which will help standardize pre-analytic factors during blood collection, and provides improved feasibility for introduction into routine ctDNA testing in the clinic.

In support of the use of the InVision liquid biopsy platform in clinical applications, data has previously been reported demonstrating a high level of concordance between this platform and dPCR in mutations detected in 35 patients with advanced breast cancer [41]. With 100% and 96% agreement for mutation detection in *ESR1* and *PIK3CA* respectively, amplicon sequencing identified additional mutations not covered by dPCR analysis and therefore substantially more mutations per patient which have possible clinical relevance. There was 100% concordance in the detection of *HER2* amplifications when compared to IHC and/or FISH of metastatic tumors [42]. Furthermore, Fribbens et al. demonstrated good concordance of eTAm-Seq technology with dPCR, with high levels of genetic heterogeneity and frequent sub-clonal mutations in advanced breast cancer patients progressing on first-line aromatase inhibitor therapy [42]. In this study, *ESR1* mutations were detectable in plasma median of 6.7 months before clinical progression. Another study compared the detection of mutations in *EGFR* between plasma and tissue and across platforms, and found amplicon-based plasma NGS to have exquisite sensitivity and specificity, with excellent quantitative concordance with an optimized dPCR assay [43]. Remon et al. have previously demonstrated the use of eTAm-Seq technology to aid in selection of targeted treatment in a prospective cohort of 48 *EGFR*-mutant advanced NSCLC patients with acquired resistance to *EGFR* TKIs, and without an available tissue biopsy [15]. cfDNA analysis identified resistance mutations in *EGFR* T790M at frequencies as low as 0.1% AF, and the study was able to demonstrate the benefit of osimertinib treatment in these patients. Strikingly, of the seven cases in that study with best response (decrease of 50% or more in size), three cases had T790M detected at <0.25% AF. Use of a less sensitive assay would miss such low frequency alleles.

Taken together, these studies demonstrate that the InVision liquid biopsy platform is a highly sensitive, quantitative and reproducible platform for detection of low frequency clinically-relevant cancer mutations in cell-free DNA. Additional larger cohorts are currently being analyzed to support clinical validation and clinical utility of the test and provide evidence to support introduction into routine testing for patient management.

## Supporting information

S1 Fig **A-E dPCR data plots for analysis of full-process control samples.** (A-D) dPCR 2D data plots for A. *EGFR* L858R/WT, B. *KRAS* G12D/WT, C. *NRAS* A59T/WT and D. *PIK3CA* E545K/WT assays showing negative controls (plasma only, NTCs), positive controls (non-spiked Multiplex I cfDNA Reference Standards) and analysis of full-process controls (plasma spiked with Multiplex I cfDNA Reference Standards). Data was analysed using QuantaLife (Bio-Rad, version 1.6.6.0320) with classification of single positive (mutant (blue), wild-type (green)), double positive (orange) and negative (black) droplets by manual crosshair setting (A, B, D) or lasso (C; lassos not shown by software post-analysis). E. Accepted droplet number for all four dPCR assays.
(EPS)

S2 Fig Sensitivity of the eTAm-Seq technology. (AI)

S3 Fig Evaluation of extraction efficiency using full-process control samples. Copy number concentrations of each target in spiked extracts were quantified by dPCR and extraction efficiency calculated by comparison with the values assigned by dPCR using the same assay.
(AI) Click here for additional data file.

S4 Fig Precision of eTAm-Seq technology and dPCR measurements of full-process control materials. Precision is expressed as % coefficient of variation (%CV) for measurements of full-process control materials spiked with 5% and 1% AF Multiplex I cfDNA Reference Standards (n = 5 (5% Reference Standard); n = 6 (1% Reference Standard) and reflects both extraction and analytical variability.
(AI) Click here for additional data file.

S5 Fig Analytical sensitivity of eTAm-Seq technology and dPCR measurements of full-process control materials. Detection rate of mutant targets by eTAm-Seq technology and dPCR in full-process control materials spiked with 5%, 1% and 0.1% AF Multiplex I cfDNA Reference Standards expressed as number of positive measurements (n = 6).
(AI) Click here for additional data file.

S1 File dMIQE checklist. (PDF)

S2 File dPCR assay validation for dMIQE review. (PDF)

S1 Table Exonic regions covered by the InVision liquid biopsy tumor profiling panel. (XLSX)

S2 Table **A-C. A. dPCR assay information B. dPCR assay information (custom design) C. PCR cycling conditions**.
(DOCX)

S3 Table LOD of dPCR assays. (DOCX)

S4 Table Table of allele fractions (shown as percentages) for the 32 common low frequency variants validated/quantified in Horizon Tru-Q 7 and in the InVision liquid biopsy panel for both laboratories (one tab per laboratory). Rows list all combination of variants (32 variants) and dilution levels (5 levels). Columns show the operator/repeats. The last column indicates the expected allele fraction based on dPCR data supplied by Horizon Discovery, and the dilution level. Missing values indicate that no call was made for that combination of variant/dilution/operator/repeat.
(XLSX)

S5 Table List of variants analyzed in the Horizon Tru-Q 6 and Horizon Tru-Q 7 dilution study. (XLSX)

S6 Table Sensitivity analysis for all samples. (XLSX)

S7 Table Table of all reportable calls by eTAm-Seq technology using the novel full-process control material. (XLSX)

## References

[1] Stroun M, Lyautey J, Lederrey C, Olson-Sand A, Anker P. About the possible origin and mechanism of circulating DNA apoptosis and active DNA release. Clin Chim Acta. 2001 11;313(1–2):139–42. 11694251 10.1016/s0009-8981(01)00665-9

[2] Sidransky D, Von Eschenbach A, Tsai YC, Jones P, Summerhayes I, Marshall F, et al Identification of p53 gene mutations in bladder cancers and urine samples. Science. 1991 5 3;252(5006):706–9. 2024123 10.1126/science.2024123

[3] Wan JC, Massie C, Garcia-Corbacho J, Mouliere F, Brenton JD, Caldas C, et al Liquid biopsies come of age: towards implementation of circulating tumour DNA. Nat Rev Cancer. 2017 4;17(4):223–238. doi: 10.1038/nrc.2017.7 28233803 10.1038/nrc.2017.7

[4] Diehl F, Schmidt K, Choti MA, Romans K, Goodman S, Li M, et al Circulating mutant DNA to assess tumor dynamics. Nat Med. 2008; 14(9):985–90. doi: 10.1038/nm.1789 18670422 10.1038/nm.1789 PMC2820391

[5] Dawson SJ, Tsui DW, Murtaza M, Biggs H, Rueda OM, Chin SF, et al Analysis of circulating tumor DNA to monitor metastatic breast cancer. N Engl J Med. 2013 3 28; 368(13):1199–209. doi: 10.1056/NEJMoa1213261 23484797 10.1056/NEJMoa1213261

[6] Murtaza M, Dawson SJ, Tsui DW, Gale D, Forshew T, Piskorz AM, et al Non-invasive analysis of acquired resistance to cancer therapy by sequencing of plasma DNA. Nature. 2013 5 2;497(7447):108–12. doi: 10.1038/nature12065 23563269 10.1038/nature12065

[7] Parkinson CA, Gale D, Piskorz AM, Biggs H, Hodgkin C, Addley H, et al Exploratory Analysis of TP53 Mutations in Circulating Tumour DNA as Biomarkers of Treatment Response for Patients with Relapsed High-Grade Serous Ovarian Carcinoma: A Retrospective Study. PLoS Med. 2016 12 20; 13(12):e1002198 doi: 10.1371/journal.pmed.1002198 27997533 10.1371/journal.pmed.1002198 PMC5172526

[8] Mok TS, Wu YL, Thongprasert S, Yang CH, Chu DT, Saijo N, et al Gefitinib or carboplatin-paclitaxel in pulmonary adenocarcinoma. N Engl J Med. 2009 9 3;361(10):947–57. doi: 10.1056/NEJMoa0810699 19692680 10.1056/NEJMoa0810699

[9] Rosell R, Carcereny E, Gervais R, Vergnenegre A, Massuti B, Felip E, et al Erlotinib versus standard chemotherapy as first-line treatment for European patients with advanced EGFR mutation-positive non-small-cell lung cancer (EURTAC): a multicentre, open-label, randomised phase 3 trial. Lancet Oncol. 2012 3;13(3):239–46. Epub 2012 Jan 26. doi: 10.1016/S1470-2045(11)70393-X 22285168 10.1016/S1470-2045(11)70393-X

[10] Cappuzzo F, Ciuleanu T, Stelmakh L, Cicenas S, Szczésna A, Juhász E, et al Erlotinib as maintenance treatment in advanced non-small-cell lung cancer: a multicentre, randomised, placebo-controlled phase 3 study. Lancet Oncol. 2010 6;11(6):521–9. Epub 2010 May 20. doi: 10.1016/S1470-2045(10)70112-1 20493771 10.1016/S1470-2045(10)70112-1

[11] McArthur GA, Chapman PB, Robert C, Larkin J, Haanen JB, Dummer R, et al Safety and efficacy of vemurafenib in BRAF(V600E) and BRAF(V600K) mutation-positive melanoma (BRIM-3): extended follow-up of a phase 3, randomised, open-label study. Lancet Oncol. 2014 3;15(3):323–32. Epub 2014 Feb 7. doi: 10.1016/S1470-2045(14)70012-9 24508103 10.1016/S1470-2045(14)70012-9 PMC4382632

[12] Diaz LA Jr, Williams RT, Wu J, Kinde I, Hecht JR, Berlin J, et al The molecular evolution of acquired resistance to targeted EGFR blockade in colorectal cancers. Nature. 2012 6 28;486(7404):537–40. doi: 10.1038/nature11219 22722843 10.1038/nature11219 PMC3436069

[13] Misale S, Yaeger R, Hobor S, Scala E, Janakiraman M, Liska D, et al Emergence of KRAS mutations and acquired resistance to anti-EGFR therapy in colorectal cancer. Nature. 2012 6 28;486(7404):532–6. doi: 10.1038/nature11156 22722830 10.1038/nature11156 PMC3927413

[14] Mok TS, Wu Y-L, Ahn M-J, Garassino MC, Kim HR, Ramalingam SS, et al Osimertinib or Platinum-Pemetrexed in EGFR T790M-Positive Lung Cancer. N Engl J Med. 2017 2 16;376(7):629–640. doi: 10.1056/NEJMoa1612674 27959700 10.1056/NEJMoa1612674 PMC6762027

[15] Remon J, Caramella C, Jovelet C, Lacroix L, Lawson A, Smalley S, et al Osimertinib benefit in EGFR-mutant NSCLC patients with T790M-mutation detected by circulating tumour DNA. Ann Oncol. 2017 4 1;28(4):784–790. doi: 10.1093/annonc/mdx017 28104619 10.1093/annonc/mdx017

[16] Garcia-Murillas I, Schiavon G, Weigelt B, Ng C, Hrebien S, Cutts RJ, Cheang M, et al Mutation tracking in circulating tumor DNA predicts relapse in early breast cancer. Sci Transl Med. 2015 8 26;7(302):302ra133 doi: 10.1126/scitranslmed.aab0021 26311728 10.1126/scitranslmed.aab0021

[17] Abbosh C, Birkbak NJ, Wilson GA, Jamal-Hanjani M, Constantin T, Salari R, et al Phylogenetic ctDNA analysis depicts early-stage lung cancer evolution. Nature. 2017 4 26;545(7655):446–451. doi: 10.1038/nature22364 28445469 10.1038/nature22364 PMC5812436

[18] Bettegowda C, Sausen M, Leary RJ, Kinde I, Wang Y, Agrawal N, et al Detection of circulating tumor DNA in early- and late-stage human malignancies.; Sci Transl Med. 2014 2 19;6(224):224ra24 doi: 10.1126/scitranslmed.3007094 24553385 10.1126/scitranslmed.3007094 PMC4017867

[19] Vogelstein B, Kinzler KW. Digital PCR. Proc Natl Acad Sci U S A. 1999 8 3;96(16):9236–41. 10430926 10.1073/pnas.96.16.9236 PMC17763

[20] US Food & Drug Administration. Premarket approval P150044, Cobas EGFR mutation test V2. 2017 FDA Available from: http://www.accessdata.fda.gov/scripts/cdrh/cfdocs/cfpma/pma.cfm?id=P150044

[21] US Food & Drug Administration. Device Approvals and Clearances. Cobas® EGFR Mutation Test v2—P150047. Patient Labeling. Table 22. Available from https://www.accessdata.fda.gov/cdrh_docs/pdf15/P150044C.pdf

[22] Forshew T, Murtaza M, Parkinson C, Gale D, Tsui DW, Kaper F, et al Noninvasive identification and monitoring of cancer mutations by targeted deep sequencing of plasma DNA. Sci Transl Med. 2012 5 30;4(136):136ra68 doi: 10.1126/scitranslmed.3003726 22649089 10.1126/scitranslmed.3003726

[23] Newman AM, Bratman SV, To J, Wynne JF, Eclov NC, Modlin LA, et al An ultrasensitive method for quantitating circulating tumor DNA with broad patient coverage. Nat Med. 2014 5;20(5):548–54 doi: 10.1038/nm.3519 24705333 10.1038/nm.3519 PMC4016134

[24] Lanman RB, Mortimer SA, Zill OA, Sebisanovic D, Lopez R, Blau S, et al Analytical and Clinical Validation of a Digital Sequencing Panel for Quantitative, Highly Accurate Evaluation of Cell-Free Circulating Tumor DNA. PLoS One. 2015 10 16;10(10):e0140712 doi: 10.1371/journal.pone.0140712 26474073 10.1371/journal.pone.0140712 PMC4608804

[25] Gale D, Plagnol V, Lawson A, Pugh M, Smalley S, Howarth K, et al. Cancer Research, AACR 2016, Abstract 3639: Analytical performance and validation of an enhanced TAm-Seq circulating tumor DNA sequencing assay; doi: 10.1158/1538-7445.AM2016-3639 Published July 2016

[26] Rosenfeld N., Forshew, T., Marass, F. & Murtaza, M. A method for detecting a genetic variant. World Intellectual Property Organization patent WO2016009224A1 (2016).

[27] McLaren W, Gil L, Hunt SE, Singh Riat H, Ritchie GRS, Thormann A, et al The Ensembl Variant Effect Predictor. Genome Biology. 2016 6 6;17(1):122 doi: 10.1186/s13059-016-0974-4 27268795 10.1186/s13059-016-0974-4 PMC4893825

[28] Whale AS, Bushell CA, Grant PR, Cowen S, Gutierrez-Aguirre I, O'Sullivan DM, et al Detection of Rare Drug Resistance Mutations by Digital PCR in a Human Influenza A Virus Model System and Clinical Samples. J Clin Microbiol. 2016 2;54(2):392–400. Epub 2015 Dec 9. doi: 10.1128/JCM.02611-15 26659206 10.1128/JCM.02611-15 PMC4733194

[29] Phallen J, Sausen M, Adleff V, Leal A, Hruban C, et al Direct detection of early-stage cancers using circulating tumor DNA. Sci Transl Med. 2017 8 16;9(403). pii: eaan2415. doi: 10.1126/scitranslmed.aan2415 10.1126/scitranslmed.aan2415 PMC6714979 28814544

[30] Devonshire AS, Whale AS, Gutteridge A, Jones G, Cowen S, Foy CA, et al Towards standardisation of cell-free DNA measurement in plasma: controls for extraction efficiency, fragment size bias and quantification. Anal Bioanal Chem. 2014 10;406(26):6499–512. doi: 10.1007/s00216-014-7835-3 24853859 10.1007/s00216-014-7835-3 PMC4182654

[31] 209.US National Library of Medicine. ClinicalTrials.gov Available from: https://clinicaltrials.gov/ct2/show/NCT02284633 (2015).

[32] 210, US National Library of Medicine. ClinicalTrials.gov Available from: https://clinicaltrials.gov/ct2/show/NCT02743910 (2016).

[33] 211. International Standard Randomised Controlled Trials Number Registry. isrctn.com Available from: http://www.isrctn.com/ISRCTN16945804 (2016).

[34] Oxnard GR, Paweletz CP, Kuang Y, Mach SL, O'Connell A, Messineo MM, et al Noninvasive detection of response and resistance in EGFR-mutant lung cancer using quantitative next-generation genotyping of cell-free plasma DNA. Clin Cancer Res. 2014 3 15;20(6):1698–1705. Epub 2014 Jan 15. doi: 10.1158/1078-0432.CCR-13-2482 24429876 10.1158/1078-0432.CCR-13-2482 PMC3959249

[35] Hrebien S, O'Leary B, Beaney M, Schiavon G, Fribbens C, Bhambra A, et al Reproducibility of Digital PCR Assays for Circulating Tumor DNA Analysis in Advanced Breast Cancer. PLoS One. 2016 10 19;11(10): e0165023 doi: 10.1371/journal.pone.0165023 27760227 10.1371/journal.pone.0165023 PMC5070760

[36] Aigrain L, Gu Y, Quail MA. Quantitation of next generation sequencing library preparation protocol efficiencies using droplet digital PCR assays—a systematic comparison of DNA library preparation kits for Illumina sequencing. BMC Genomics. 2016 6 13;17:458 doi: 10.1186/s12864-016-2757-4 27297323 10.1186/s12864-016-2757-4 PMC4906846

[37] Krimmel JD, Schmitt MW, Harrell MI, Agnew KJ, Kennedy SR, Emond MJ, et al Ultra-deep sequencing detects ovarian cancer cells in peritoneal fluid and reveals somatic TP53 mutations in noncancerous tissues. Proc Natl Acad Sci U S A. 2016 5 24;113(21):6005–10. Epub 2016 May 5. doi: 10.1073/pnas.1601311113 27152024 10.1073/pnas.1601311113 PMC4889384

[38] Genovese G, Kähler AK, Handsaker RE, Lindberg J, Rose SA, Bakhoum SF, et al Clonal hematopoiesis and blood-cancer risk inferred from blood DNA sequence. N Engl J Med. 2014 12 25;371(26):2477–87. Epub 2014 Nov 26. doi: 10.1056/NEJMoa1409405 25426838 10.1056/NEJMoa1409405 PMC4290021

[39] Costello M, Pugh TJ, Fennell TJ, Stewart C, Lichtenstein L, Meldrim JC, et al Discovery and characterization of artifactual mutations in deep coverage targeted capture sequencing data due to oxidative DNA damage during sample preparation. Nucleic Acids Res. 2013 4 1;41(6): e67 doi: 10.1093/nar/gks1443 23303777 10.1093/nar/gks1443 PMC3616734

[40] Park G, Park JK, Shin SH, Jeon HJ, Kim NKD, Kim YJ, et al Characterization of background noise in capture-based targeted sequencing data. Genome Biol. 2017 7 21;18(1):136 doi: 10.1186/s13059-017-1275-2 28732520 10.1186/s13059-017-1275-2 PMC5521083

[41] Garcia-Murillas I, Beaney M, Epstein M, Howarth K, Lawson A, Hrebien S, et al Comparison of enhanced Tagged-Amplicon Sequencing and digital PCR for circulating tumor DNA analysis in advanced breast cancer. Cancer Res 2017;77(13 Suppl): Abstract nr 2743. doi: 10.1158/1538-7445.AM2017-2743 http://www.inivata.com/wp-content/uploads/2017/04/AACR-2017-Inivata-FINAL.pdf (AACR 2017).

[42] Fribbens C, Garcia Murillas I, Beaney M, Hrebien S, O'Leary B, Kilburn L, et al Tracking evolution of aromatase inhibitor resistance with circulating tumour DNA analysis in metastatic breast cancer. Ann Oncol. 2017 10 4 doi: 10.1093/annonc/mdx483. 10.1093/annonc/mdx483 PMC6264798 29045530

[43] Guibert NM, Paweletz C, Hu Y, Feeney NB, Plagnol V, Poole V, et al J Clin Oncol 35, 2017 (suppl; abstr 11529). doi: 10.1200/JCO.2017.35.15_suppl.11529 Journal of Clinical Oncology 35, no. 15_suppl (May 2017) 11529–11529. https://www.inivata.com/wp-content/uploads/2017/06/Guibert_ASCO.pdf (ASCO 2017).

