# Supplementary material for: Development of a highly sensitive liquid biopsy platform to detect clinically-relevant cancer mutations at low allele fractions in cell-free DNA
Source: PLoS One. 2018 Mar 16;13(3):e0194630. doi: 10.1371/journal.pone.0194630 (PMC5856404; doi:10.1371/journal.pone.0194630)
Supplement: S1 File — (PDF) [file pone.0194630.s006.pdf]

Table S6. dMIQE checklist for authors, reviewers and editors

ITEM TO CHECK	IMPORTANCE	CHECKLIST
**EXPERIMENTAL DESIGN**		
Definition of experimental and control groups	**E**	Methods
Number within each group	**E**	Methods
Assay carried out by core lab or investigator's lab?	D	LGC
Power analysis	D	ND
**SAMPLE**		
Description	**E**	Methods
Volume/mass of sample processed	D	Methods
Microdissection or macrodissection	**E**	N/A
Processing procedure	**E**	Methods (plasma homogenisation)
If frozen - how and how quickly?	**E**	Methods
If fixed - with what, how quickly?	**E**	N/A
Sample storage conditions and duration (especially for FFPE samples)	**E**	-80 °C, 6 weeks after preparation of pooled plasma
**NUCLEIC ACID EXTRACTION**		
Quantification - instrument/method	**E**	N/A (dPCR only)
DNA or RNA quantification	**E**	DNA
Quality/Integrity, method/instrument, e.g. RNA integrity	**E**	See sheet "Bioanalyzer"
Template structural information	**E**	N/A
Template modification (digestion, sonication, preamplification etc)	**E**	N/A
Template treatment (initial heating or chemical denaturation)	**E**	N/A
Inhibition dilution or spike	**E**	See Sheet "Inhibition testing"
DNA contamination assessment of RNA samples	**E**	N/A
Details of DNase treatment where performed	**E**	N/A
Manufacturer of reagents used and catalogue number	D	Qiagen, part number: 55114
Storage of nucleic acid (temperature, concentration, duration and buffer)	**E**	Samples for dPCR stored undiluted in aliquots at -20 °C for max. of 1 month
**REVERSE TRANSCRIPTION (if necessary)**		
cDNA priming method and concentration	**E**	N/A
One or two-step protocol	**E**	N/A
Amount of RNA used per reaction	**E**	N/A
Detailed reaction components and conditions	**E**	N/A
RT efficiency	D	N/A
Estimated copies measured with and without addition of RT*	D	N/A
Manufacturer of reagents and catalogue numbers	D	N/A
Reaction volume	D	N/A
Storage conditions of cDNA (temperature, concentration, duration and buffer)	D	N/A
**dPCR TARGET INFORMATION**		
Sequence accession number	**E**	Supplementary information (COSMIC ID)
Amplicon location	D	
Amplicon length	**E**	Supplementary information
In silico specificity screen (BLAST, etc)	**E**	See file "dPCR assay validation"
Pseudogenes, retropseudogenes or other homologs?	D	See file "dPCR assay validation"
Sequence alignment	D	See file "dPCR assay validation"
Secondary structure analysis of amplicon and GC content	D	ND
Location of each primer by exon or intron (if applicable)	**E**	N/A
Where appropriate, which splice variants are targeted?	**E**	N/A
**dPCR OLIGONUCLEOTIDES**		
Primer sequences and/or amplicon context sequence**	**E**	Supplementary information
RTPrimerDB Identification Number	D	ND
Probe sequences**	D	Supplementary information
Location and identity of any modifications	**E**	Supplementary information
Manufacturer of oligonucleotides	D	Methods
Purification method	D	Primers: Reverse Phase Cartridge; Probes: HPLC
**dPCR PROTOCOL**		
Complete reaction conditions	**E**	Methods
Reaction volume and amount of cDNA/DNA	**E**	Methods
Primer, (probe), Mg++ and dNTP concentrations	**E**	Supplementary information
Polymerase identity and concentration	**E**	Methods
Buffer/kit identity and manufacturer	**E**	
Exact chemical constitution of the buffer	D	Not available (Proprietary)
Additives (SYBR Green I, DMSO, etc.)	**E**	Not available (Proprietary)
Plates/tubes catalogue number and manufacturer	D	Eppendorf™ twin.tec™ PCR Plates; Fisher Scientific; 10767294
Complete thermocycling parameters	**E**	Supplementary information
Reaction setup (manual/robotic)	D	Manual droplet generation with DG8
Gravimetric or volumetric dilutions (manual/robotic)	D	Plasma extracts analysed without dilution
Total PCR volume prepared	D	22 µl
Partition number	**E**	Mean 15282 ± SD 1683
Individual partition volume	**E**	0.85 nL
Total volume of the partitions measured (effective reaction size)	**E**	Mean 13 µl (to 1 d.p.)
Partition volume variance/SD	D	ND
Comprehensive details and appropriate use of controls	**E**	Supplementary information
Manufacturer of dPCR instrument	**E**	Methods
**dPCR VALIDATION**		
Optimisation data for the assay	D	See file "dPCR assay validation"
Specificity (when measuring rare mutations, pathogen sequences etc)	**E**	See file "dPCR assay validation"
Limit of detection of calibration control	D	Supplementary information
If multiplexing, comparison with singleplex assays	**E**	N/A
**DATA ANALYSIS**		
Mean copies per partition (λ or equivalent)	**E**	See "dPCR data" sheet in this file
dPCR analysis program (source, version)	**E**	Methods
Outlier identification and disposition	**E**	1 extract (#30) removed from analysis due to low yield (WT DNA) (<50% of value for other spiked samples)
Results of NTCs	**E**	See "dPCR data" sheet in this file
Examples of positive(s) and negative experimental results as supplemental data	**E**	Supplementary information
Where appropriate, justification of number and choice of reference genes	**E**	N/A
Where appropriate, description of normalization method	**E**	N/A
Number and concordance of biological replicates	D	N/A (technical replicates at extraction stage presented in Figures and Supplementary Figures)
Number and stage (RT or qPCR) of technical replicates	**E**	n = 1 dPCR replicate per extract per target; n = 3 controls (non-spiked DNA)
Repeatability (intra-assay variation)	**E**	See "Assay repeatability" sheet in this file
Reproducibility (inter-assay/user/lab etc variation)	D	Available upon request
Experimental variance or CI***	**E**	Supplementary Figures (precision for replicate extractions)
Statistical methods for analysis	**E**	Precision metrics (SD) calculated using Microsoft Excel
Data submission using RDML (Real-time PCR Data Markup Language)	D	

All essential information (E) must be submitted with the manuscript. Desirable information (D) should be submitted if possible.

* Assessing the absence of DNA using a no RT assay (or where RT has been inactivated) is essential when first extracting RNA. Once the sample has been validated as DNA-free, inclusion of a no-RT control is desirable, but no longer essential.

** Disclosure of the primer and probe sequence is highly desirable and strongly encouraged. However, since not all commercial pre-designed assay vendors provide this information when it is not available assay context sequences must be submitted.

*** When single dPCR experiments are performed, the variation due to counting error alone

dMIQE Information: DNA quality: plasma cfDNA extracts

cfDNA isolated from pooled plasma (not spiked with Horizon cfDNA Reference Standards) was analysed using the Agilent 2100 Bioanalyzer with the High Sensitivity DNA Assay version 1.03.

Extracts (1-9) show typical pattern for cfDNA consisting of ~ 180 bp peak and longer (>1 kb) molecular weight DNA.
(Samples 10 and 11 are replicates of the size ladder provided with the kit).

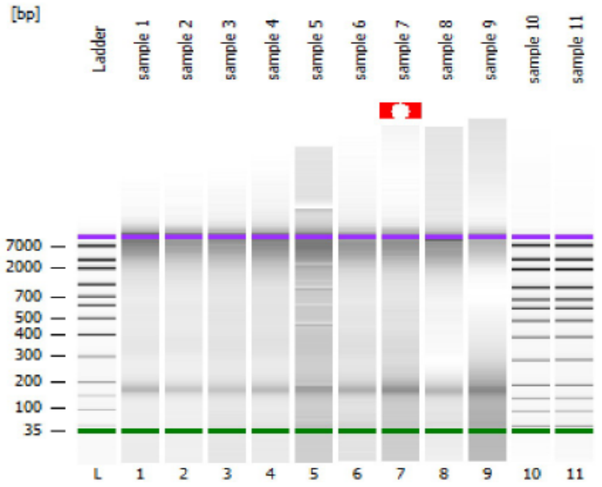

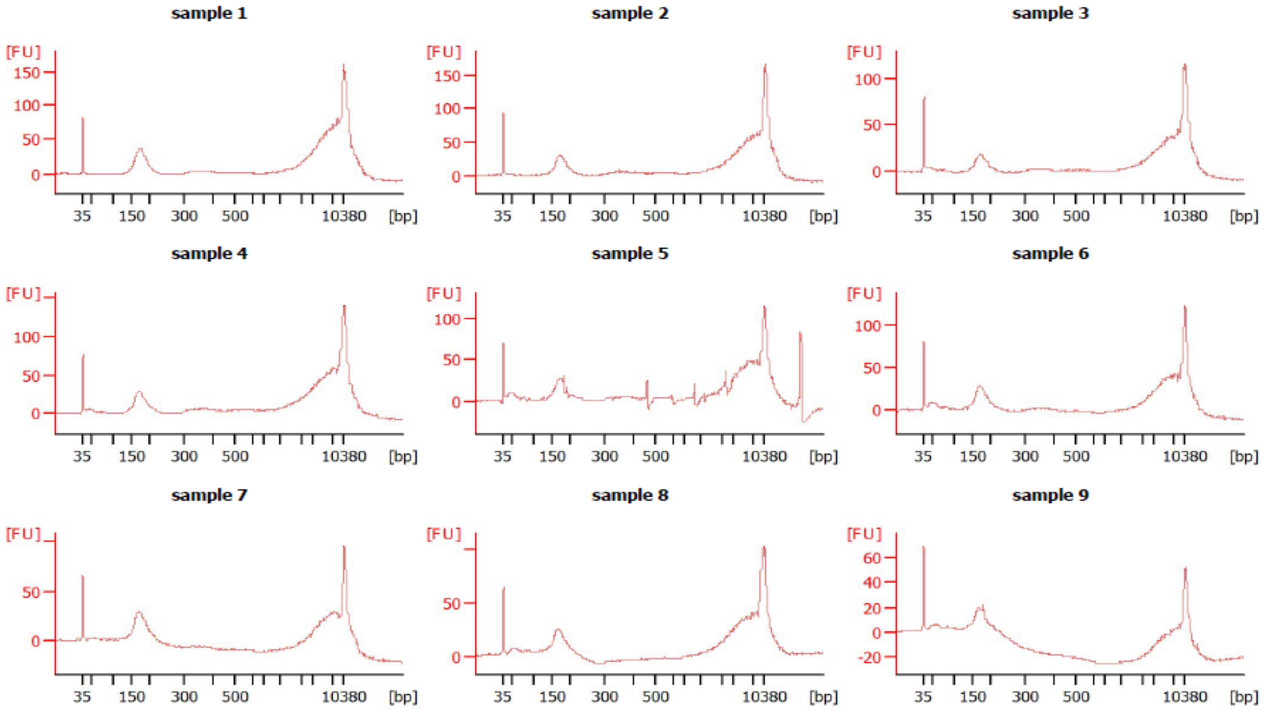

dMIQE information: Assay inhibition testing: testing for inhibition by plasma cfDNA extracts

8 ng Horizon gDNA Reference Standards (Table 1) were added per 20 µL dPCR assay with/without 5 µl of plasma extract from unspiked plasma (prepared as in Materials and Methods).

Table: Control materials using for inhibition testing

Gene	Variant	Cat No	Lot	% Mutant AF
**EGFR**	**L858R**	**HD-C885**	**15664**	50%
**KRAS**	**G12D**	**HD-C889**	**15668**	50%
**NRAS**	**E545K**	**HD697**	**11263**	50%
**PI3KCA**	**A59T**	**HD689**	**11826**	50%

No difference in the quantity of the mutant allele was observed in the reactions spiked with plasma extract (Figure).

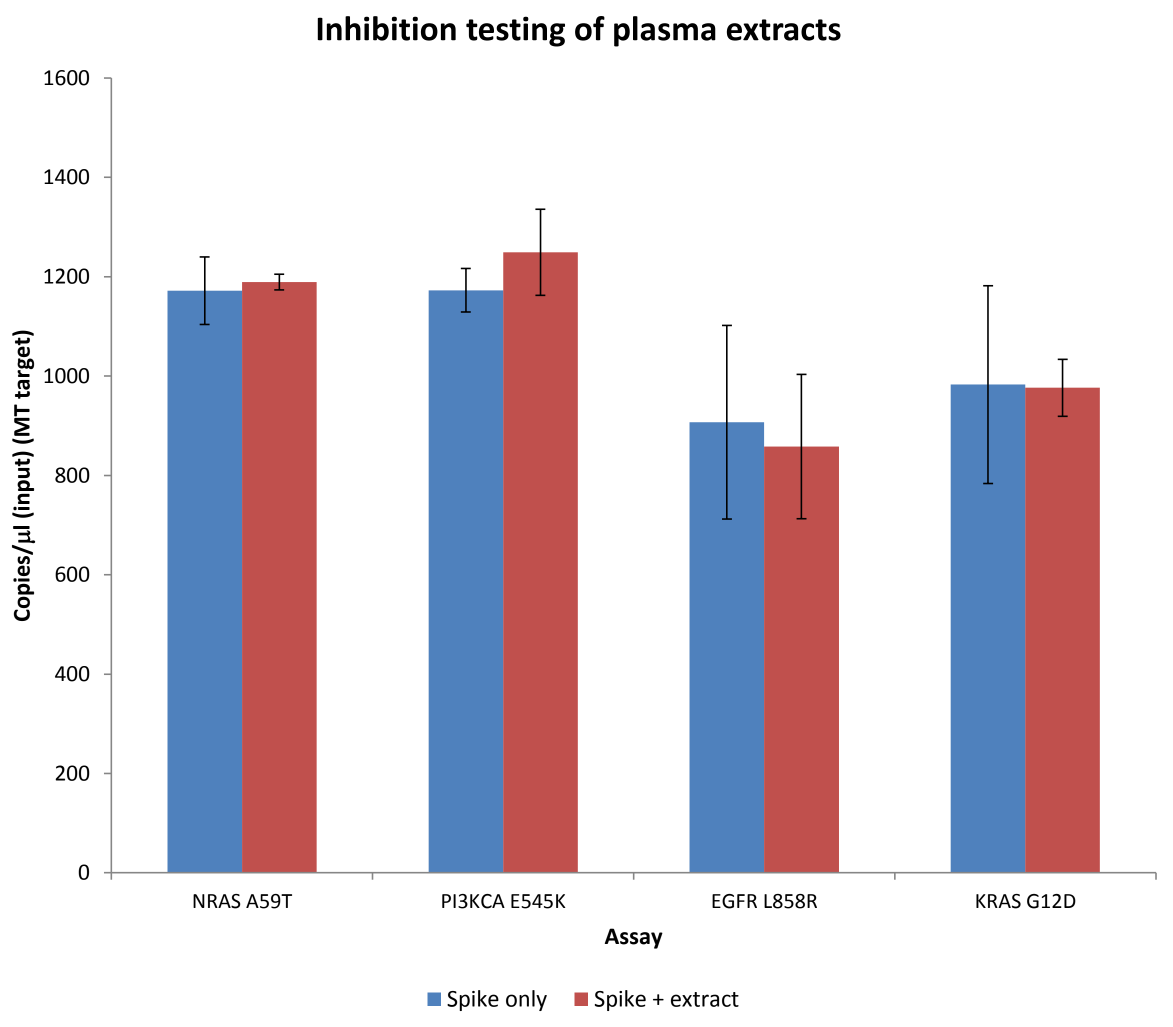

dMIQE information: Experimental dPCR data

Sample Number	Spike mAF	NRAS A59T				KRAS G12D				EGFR L858R				PIK3CA E545K			
		k mt	k wt	n	λ	k mt	k wt	n	λ	k mt	k wt	n	λ	k mt	k wt	n	λ
**1**	Plasma only	0	250	12932	0.00000	0	330	14618	0.00000	0	364	13900	0.00000	0	284	12417	0.00000
**2**	5.00%	67	1396	12115	0.006	117	2230	15362	0.008	75	1923	12483	0.006	62	1373	11944	0.005
**3**	1.00%	18	1687	13522	0.001	22	2225	14356	0.002	15	2012	11481	0.001	13	1570	12456	0.001
**4**	0.10%	1	2063	13316	0.00008	6	3237	15988	0.0004	0	2764	13038	0.00000	4	2048	12431	0.0003
**5**	0.00%	1	1318	12514	0.00008	0	1935	14447	0.00000	0	1922	13163	0.00000	1	1616	14686	0.00007
**6**	Plasma only	0	423	12707	0.00000	0	580	14845	0.00000	0	533	12580	0.00000	0	480	13076	0.00000
**7**	5.00%	80	1492	14973	0.005	104	2087	15625	0.007	70	1741	12077	0.006	78	1666	15117	0.005
**8**	1.00%	23	2382	15282	0.002	35	3042	15781	0.002	24	2935	14283	0.002	14	1157	7633	0.002
**9**	0.10%	4	1568	15144	0.0003	4	2042	13983	0.0003	0	1991	14224	0.0000	5	1577	14450	0.0003
**10**	0.00%	0	1245	13417	0.00000	1	1776	15653	0.00006	0	1640	12578	0.00000	0	1444	15337	0.00000
**11**	0.10%	4	2336	15185	0.0003	1	3827	17686	0.0001	4	3413	14952	0.0003	6	2817	17316	0.0003
**12**	5.00%	123	2368	15071	0.008	189	3517	17751	0.01	118	3054	13828	0.009	149	2688	16202	0.009
**13**	0.10%	4	2389	13388	0.0003	9	3039	13104	0.0007	6	2963	12113	0.0005	4	2277	12572	0.0003
**14**	0.00%	0	1574	15230	0.00000	1	2290	16883	0.00006	0	2131	14614	0.00000	0	1866	17017	0.00000
**15**	1.00%	11	1642	14483	0.0008	20	2341	15490	0.001	15	2135	13283	0.001	28	1891	16256	0.002
**16**	Plasma only	0	448	14981	0.00000	0	578	17218	0.00000	0	523	14788	0.00000	0	477	15169	0.00000
**17**	5.00%	87	2012	16208	0.005	143	2794	16957	0.008	89	2458	14126	0.006	108	2114	16364	0.007
**18**	Plasma only	0	461	13285	0.00000	0	553	14068	0.00000	0	574	13878	0.00000	0	480	13737	0.00000
**19**	0.00%	0	2791	15093	0.00000	0	3283	15755	0.00000	1	3086	12702	0.00008	0	3158	16998	0.00000
**20**	1.00%	24	1908	14593	0.002	40	2891	17107	0.002	21	2714	15316	0.001	20	2273	17046	0.001
**21**	1.00%	26	2463	15830	0.002	37	3259	17331	0.002	21	3474	16732	0.001	31	2903	15763	0.002
**22**	0.00%	0	1198	13275	0.00000	0	2229	16976	0.00000		2120	15690	0.00000	0	1808	17726	0.00000
**23**	0.10%	3	1813	14477	0.0002	3	2592	15803	0.0002	1	2666	15630	0.0001	3	2193	17678	0.0002
**24**	0.00%	0	2863	15835	0.00000	1	3541	15547	0.00006	0	3745	16125	0.00000	1	2908	15817	0.00006
**25**	Plasma only	0	654	15778	0.0000	1	858	16778	0.0001	0	752	14803	0.00000	0	807	16019	0.0000
**26**	5.00%	78	1735	15978	0.005	110	2298	15909	0.007	98	2533	15990	0.006	124	2101	18381	0.007
**27**	Plasma only	0	508	14921	0.00000	0	541	16531	0.00000	0	514	14193	0.00000	0	478	15658	0.00000
**28**	1.00%	18	2015	16293	0.001	29	2677	16394	0.002	21	2478	15033	0.001	32	2033	16229	0.002
**29**	0.10%	1	1319	14790	0.00007	1	1834	16941	0.00006	0	1544	13703	0.00000	3	1120	13405	0.00022
**30**	5.00%	38	790	16405	0.002	55	883	14613	0.004	45	1053	15707	0.003	37	763	15919	0.002
**NTC TE**	**NA**	0	0	13966	0.00000	0	1	17042	0.00000	0	0	14453	0.00000	0	0	19061	0.00000
**NTC TE**	**NA**	0	2	15829	0.00000	1	0	15577	0.00006	0	1	15069	0.00000	0	0	14575	0.00000
**NTC TE**	**NA**	0	0	15925	0.00000	0	3	16987	0.00000	0	0	14783	0.00000	0	0	19025	0.00000
**NTC H2O**	**NA**	0	0	17291	0.00000	0	2	17756	0.00000	0	0	12852	0.00000	0	0	15828	0.00000
**NTC H2O**	**NA**	0	3	17606	0.00000	0	2	15890	0.00000	0	0	17364	0.00000	0	0	17177	0.00000
**NTC H2O**	**NA**	0	1	17331	0.00000	0	4	17023	0.00000	0	0	15919	0.00000	0	0	16548	0.00000

dMIQE information: Assay repeatability

Assay performance was tested with a 5 point 5-fold dilution series of gDNA Reference Standard (50% mAF) (Horizon, P/Ns below) from 40 ng to 0.064 ng/reaction (MT1 to MT5).

Gene	Mutation	Horizon P/N
EGFR	L858R	HD-C885
KRAS	G12D	HD-C889
NRAS	A59T	HD-C891
PI3KCA	E545K	HD-C892

						Mutant concentration	Mutant Allelic Frequency (mAF) (%)				
Experimental set-up	Sample	Positive droplets	Negative droplets	Accepted droplets	λ	Input (c/µl) (5 ul sample per reaction)	Value	Poisson error	Mean	SD	%CV
Assay: EGFR L858R/WT											
ABS	MT1	1073	12123	13196	0.0848	998	50.2	2	49.4	1.00	2%
ABS	MT1	1186	14302	15488	0.0797	937	49.8	2			
ABS	MT1	1210	14542	15752	0.0799	940	48.3	2			
ABS	MT2	242	14015	14257	0.0171	201	48.5	4	48.7	1.86	4%
ABS	MT2	244	16017	16261	0.0151	178	46.9	4			
ABS	MT2	267	16075	16342	0.0165	194	50.6	4			
ABS	MT3	42	12877	12919	0.0033	38	52.0	11	49.0	7.94	16%
ABS	MT3	44	16926	16970	0.0026	31	40.0	10			
ABS	MT3	61	16793	16854	0.0036	43	55.0	9			
ABS	MT4	10	14429	14439	0.0007	8	48.0	21	48.0	4.00	8%
ABS	MT4	14	16087	16101	0.0009	10	44.0	17			
ABS	MT4	12	15508	15520	0.0008	9	52.0	21			
ABS	MT5	2	14163	14165	0.0001	2	40.0	47	53.3	15.28	29%
ABS	MT5	2	17319	17321	0.0001	1	70.0	60			
ABS	MT5	3	16525	16528	0.0002	2	50.0	43			
Assay: KRAS G12D/WT											
ABS	MT1	2132	10669	12801	0.1822	857	50.1	2	50.4	0.79	2%
ABS	MT1	2088	10656	12744	0.1789	842	49.8	2			
ABS	MT1	2730	13443	16173	0.1849	870	51.3	1			
ABS	MT2	487	13305	13792	0.0359	169	52.0	3	51.4	1.46	3%
ABS	MT2	480	13509	13989	0.0349	164	49.7	3			
ABS	MT2	584	15515	16099	0.0369	174	52.4	3			
ABS	MT3	110	15772	15882	0.0070	33	45.0	6	46.0	1.00	2%
ABS	MT3	109	15826	15935	0.0069	32	47.0	6			
ABS	MT3	108	16074	16182	0.0067	32	46.0	7			
ABS	MT4	39	14747	14786	0.0026	12	63.0	12	51.7	11.02	21%
ABS	MT4	20	15277	15297	0.0013	6	41.0	14			
ABS	MT4	24	16354	16378	0.0015	7	51.0	14			
ABS	MT5	4	15025	15029	0.0003	1	57.0	39	35.7	18.72	52%
ABS	MT5	4	15530	15534	0.0003	1	22.0	20			
ABS	MT5	5	16641	16646	0.0003	1	28.0	21			
Assay: NRAS A59T/WT											
ABS	MT1	3268	14011	17279	0.2096	987	50.2	1	49.7	0.46	1%
ABS	MT1	3307	14354	17661	0.2073	976	49.6	1			
ABS	MT1	3238	14386	17624	0.2030	955	49.3	1			
ABS	MT2	694	16807	17501	0.0405	190	46.7	3	48.1	1.31	3%
ABS	MT2	828	18863	19691	0.0430	202	49.3	2			
ABS	MT2	766	18470	19236	0.0406	191	48.2	3			
ABS	MT3	135	17042	17177	0.0079	37	45.0	6	48.3	2.89	6%
ABS	MT3	143	18069	18212	0.0079	37	50.0	5			
ABS	MT3	150	16971	17121	0.0088	41	50.0	6			
ABS	MT4	29	19496	19525	0.0015	7	49.0	13	52.0	3.00	6%
ABS	MT4	32	19465	19497	0.0016	8	55.0	13			
ABS	MT4	17	16350	16367	0.0010	5	52.0	17			
ABS	MT5	5	19436	19441	0.0003	1	36.0	26	55.0	19.52	35%
ABS	MT5	6	16201	16207	0.0004	2	75.0	33			
ABS	MT5	7	18535	18542	0.0004	2	54.0	28			
Assay: PI3KCA E545K/WT											
ABS	MT1	3098	15802	18900	0.1790	842	49.1	1	49.5	0.46	1%
ABS	MT1	3073	15562	18635	0.1802	848	50.0	1			
ABS	MT1	2888	14432	17320	0.1824	858	49.4	1			
ABS	MT2	529	16197	16726	0.0321	151	46.8	3	48.2	1.99	4%
ABS	MT2	539	16255	16794	0.0326	154	47.4	3			
ABS	MT2	675	18548	19223	0.0357	168	50.5	3			
ABS	MT3	126	17668	17794	0.0071	33	48.0	7	48.7	1.15	2%
ABS	MT3	115	16296	16411	0.0070	33	48.0	7			
ABS	MT3	154	19784	19938	0.0078	36	50.0	6			
ABS	MT4	29	19530	19559	0.0015	7	48.0	13	49.0	3.61	7%
ABS	MT4	21	16487	16508	0.0013	6	46.0	14			
ABS	MT4	42	19450	19492	0.0022	10	53.0	11			
ABS	MT5	7	19234	19241	0.0004	2	39.0	23	46.7	7.51	16%
ABS	MT5	7	18105	18112	0.0004	2	47.0	26			
ABS	MT5	7	19922	19929	0.0004	2	54.0	28			
