# Supplementary material for: Development of a highly sensitive liquid biopsy platform to detect clinically-relevant cancer mutations at low allele fractions in cell-free DNA
Source: PLoS One. 2018 Mar 16;13(3):e0194630. doi: 10.1371/journal.pone.0194630 (PMC5856404; doi:10.1371/journal.pone.0194630)
Supplement: S2 File — (PDF) [file pone.0194630.s007.pdf]

dMIQE Information: Assay validation

1. In silico specificity testing
2. dPCR assay optimisation
3. dPCR specificity testing (wet lab)

1. In silico specificity testing

- In silico specificity of primers checked using NCBI Primer Blast
- Primer and probe aligned to specific and non-specific chromosome regions using Geneious (version R8)

In silico specificity testing: NRAS A59T/WT

NCBI Primer Blast

```
>NC_000001.11 Homo sapiens chromosome 1, GRCh38.p7 Primary Assembly

product length = 89
Features associated with this product:
   GTPase NRas

Forward primer  1          GATGGTGAAACCTGTTTGTTGGA  23
Template        114713951  .......................  114713929

Reverse primer  1          TCGCCTGTCCTCATGTATTGG  21
Template        114713863  .....................  114713883

>NC_000012.12 Homo sapiens chromosome 12, GRCh38.p7 Primary Assembly

product length = 89
Features associated with this product:
   GTPase KRas isoform b

   GTPase KRas isoform X2

Forward primer  1         GATGGTGAAACCTGTTTGTTGGA  23
Template        25227385  .....A.........C.C.....  25227363


Reverse primer  1         TCGCCTGTCCTCATGTATTGG  21
Template        25227297  ..C..A...........C...  25227317

>NC_000006.12 Homo sapiens chromosome 6, GRCh38.p7 Primary Assembly

product length = 88
Features flanking this product:
   380797 bp at 5' side: tubulointerstitial nephritis antigen isoform X6
   99438 bp at 3' side: protein FAM83B isoform X1

Forward primer  1         GATGGTGAAACCTGTTTGTTGGA  23
Template        54770721  .....A.........C.C.....  54770743

Reverse primer  1         TCGCCTGTCCTCATGTATTGG  21
Template        54770808  CGC..A...........C...  54770788
```

In silico specificity testing: NRAS A59T/WT

Geneious primer and probe mapping (maximum 4 mismatches)

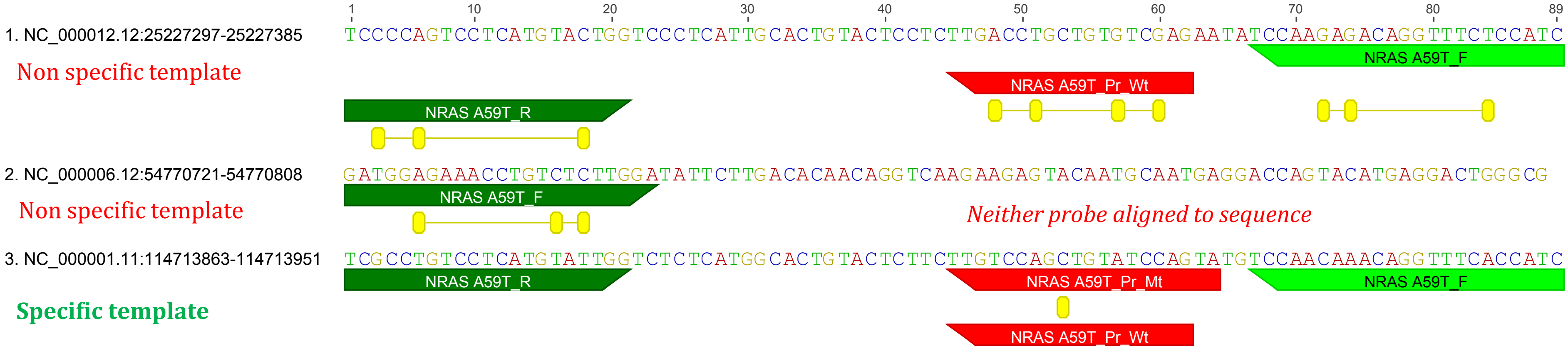

Geneious alignment of primers to reference sequence (chromosome 6)

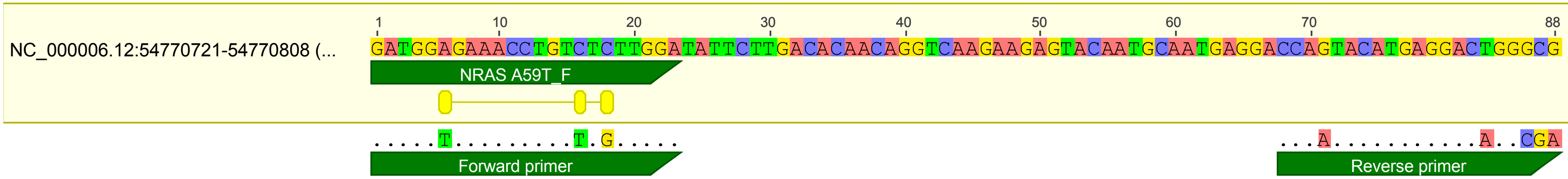

In silico specificity testing: PI3KCA E545K/WT

NCBI Primer Blast

>NC_000003.12 Homo sapiens chromosome 3, GRCh38.p7 Primary Assembly

```
product length = 89
Features associated with this product:
   phosphatidylinositol 4,5-bisphosphate 3-kinase catalytic ...

   phosphatidylinositol 4,5-bisphosphate 3-kinase catalytic ...

Forward primer  1          GAACAGCTCAAAGCAATTTCTACAC  25
Template        179218255  .........................  179218279

Reverse primer  1          AGCACTTACCTGTGACTCCATAG  23
Template        179218343  .......................  179218321
```

>NC_000022.11 Homo sapiens chromosome 22, GRCh38.p7 Primary Assembly

```
product length = 89
Features flanking this product:
   751417 bp at 5' side: uncharacterized protein LOC101929839
   18716 bp at 3' side: T-complex protein 1 subunit theta-like 2

Forward primer  1         GAACAGCTCAAAGCAATTTCTACAC  25
Template        16572073  .........................  16572097

Reverse primer  1         AGCACTTACCTGTGACTCCATAG  23

Template        16572160  ..............-G.......  16572139
```

In silico specificity testing: PI3KCA E545K/WT

Geneious primer mapping of primers and probes to PI3KCA pseudogene (maximum 4 mismatches)

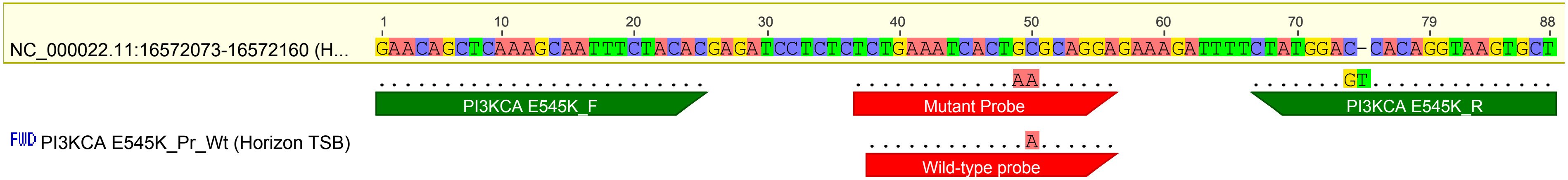

Amplification of PI3KCA pseudogene was minimised by selecting a higher annealing temperature during dPCR optimisation experiments (see Part 2 of this document).

2. dPCR assay optimisation

- In house assays to NRAS A59T and PIK3CA E545K were optimised by analysing a range of PCR annealing temperatures from 53°C - 65°C
- Horizon gDNA Reference Standards (50% mAF and 100% WT) at two concentrations were measured ($\lambda 1$ and $\lambda 2$):
  - Part number HD-C891 (50% NRAS A59T)
  - Part number HD-C892 (50% PI3KCA)
  - Part number HD-C884 (100% WT)
- The optimal annealing temperature setting is indicated by a green box

NRAS A59T FAM/Cal Fluo Orange 650 (FAM/HEX settings). Same threshold settled for each temperature

MT λ1 65.0 °C (intact; ≈ 8 ng/rxn)

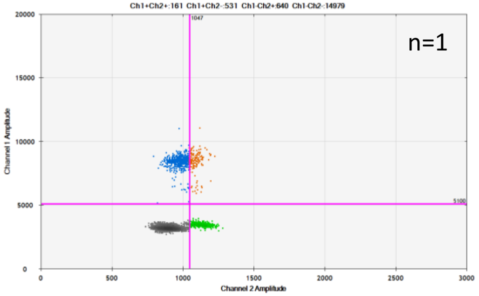

MT λ1 65.0 °C (intact; ≈ 2 ng/rxn)

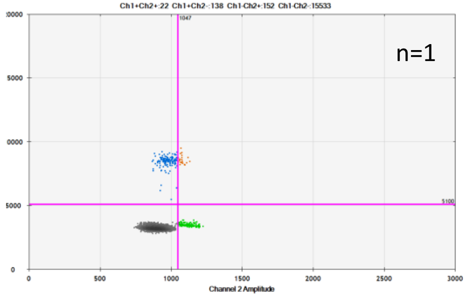

WT λ1 65.0 °C (intact; ≈ 8 ng/rxn)

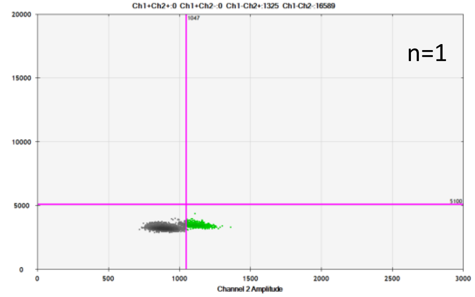

WT λ1 65.0 °C (intact; ≈ 2 ng/rxn)

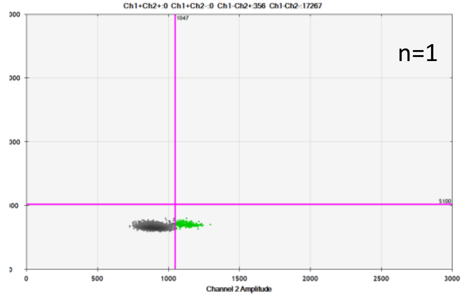

Threshold=> Ch1: 5100, Ch2: 1047

MT λ1 62.7 °C (intact; ≈ 8 ng/rxn)

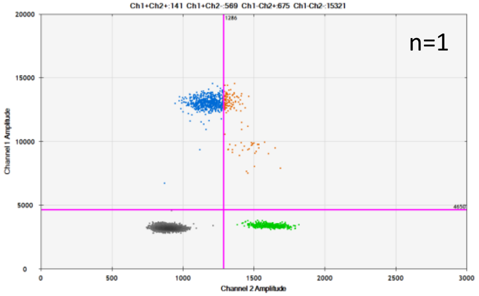

MT λ1 62.7 °C (intact; ≈ 2 ng/rxn)

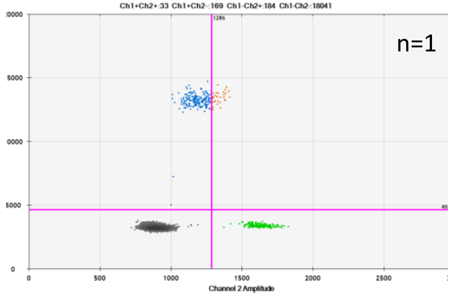

WT λ1 62.7 °C (intact; ≈ 8 ng/rxn)

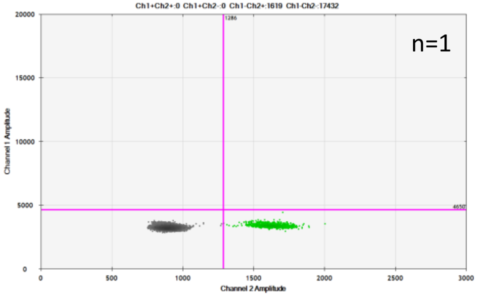

WT λ1 62.7 °C (intact; ≈ 2 ng/rxn)

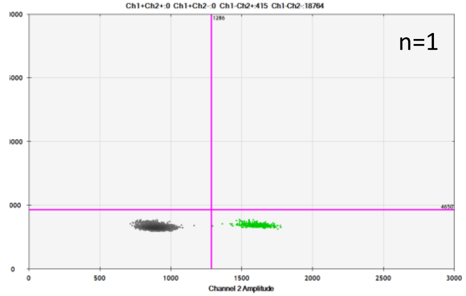

Threshold=> Ch1: 4650, Ch2: 1286

MT λ1 60.4 °C (intact; ≈ 8 ng/rxn)

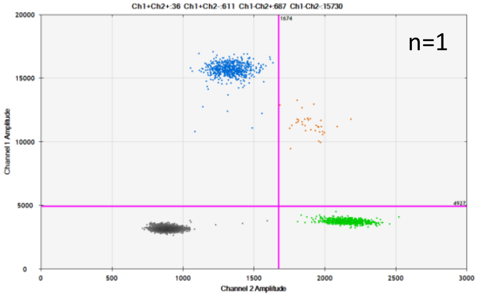

MT λ1 60.4 °C (intact; ≈ 2 ng/rxn)

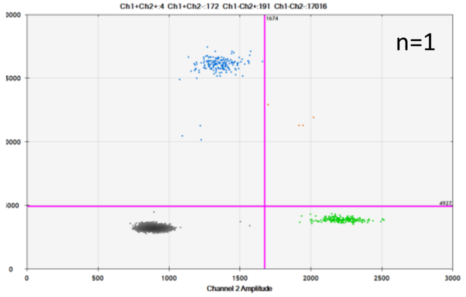

WT λ1 60.4 °C (intact; ≈ 8 ng/rxn)

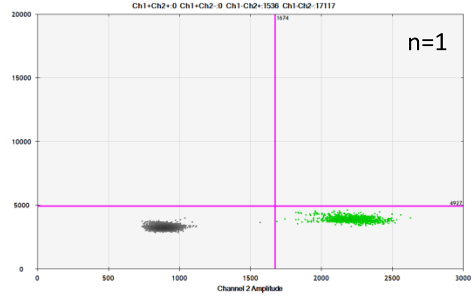

WT λ1 60.4 °C (intact; ≈ 2 ng/rxn)

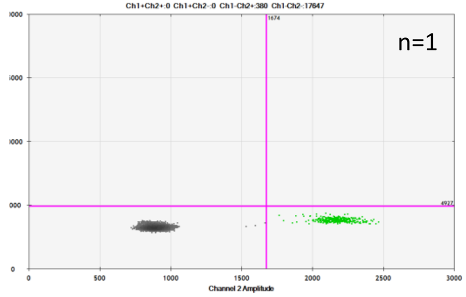

Threshold=> Ch1: 4927, Ch2: 1647

NRAS A59T FAM/Cal Fluo Orange 650 (FAM/HEX settings). Threshold settled for each temperature

MT λ1 57.8 °C (intact; ≈ 8 ng/rxn) | **MT λ1 57.8 °C** (intact; ≈ 2 ng/rxn) | **WT λ1 57.8 °C** (intact; ≈ 8 ng/rxn) | **WT λ1 57.8 °C** (intact; ≈ 2 ng/rxn)

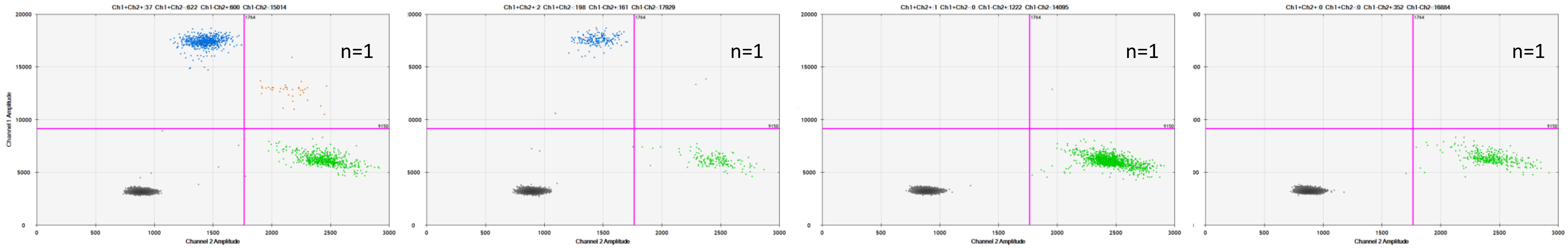

Threshold=> Ch1: 9150, Ch2: 1764

MT λ1 55.4 °C (intact; ≈ 8 ng/rxn) | **MT λ1 55.4 °C** (intact; ≈ 2 ng/rxn) | **WT λ1 55.4 °C** (intact; ≈ 8 ng/rxn) | **WT λ1 55.4 °C** (intact; ≈ 2 ng/rxn)

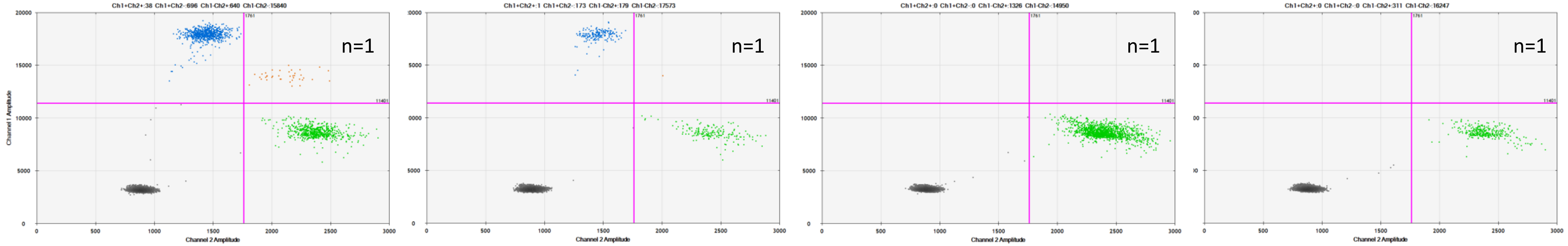

Threshold=> Ch1: 11401, Ch2: 1761

MT λ1 53.0 °C (intact; ≈ 8 ng/rxn) | **MT λ1 53.0 °C** (intact; ≈ 2 ng/rxn) | **WT λ1 53.0 °C** (intact; ≈ 8 ng/rxn) | **WT λ1 53.0 °C** (intact; ≈ 2 ng/rxn)

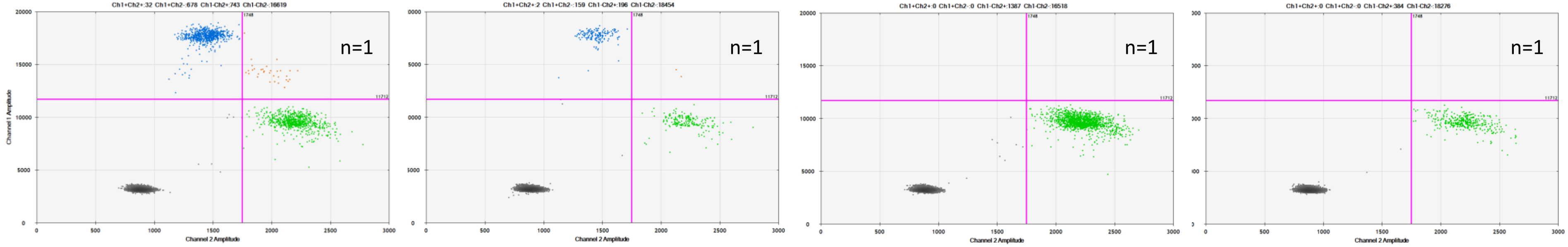

Threshold=> Ch1: 11712, Ch2: 1748

S:\Science & Technology\UK\Teddington\Programmes\CBM-2\Projects\Horizon TSB\WP3\Data\Exp053 NRAS A59T & EGRFins assay optimisation (QX200)

NRAS A59T FAM/Cal Fluo Orange 650 (FAM/HEX settings). Same threshold settled for each temperature

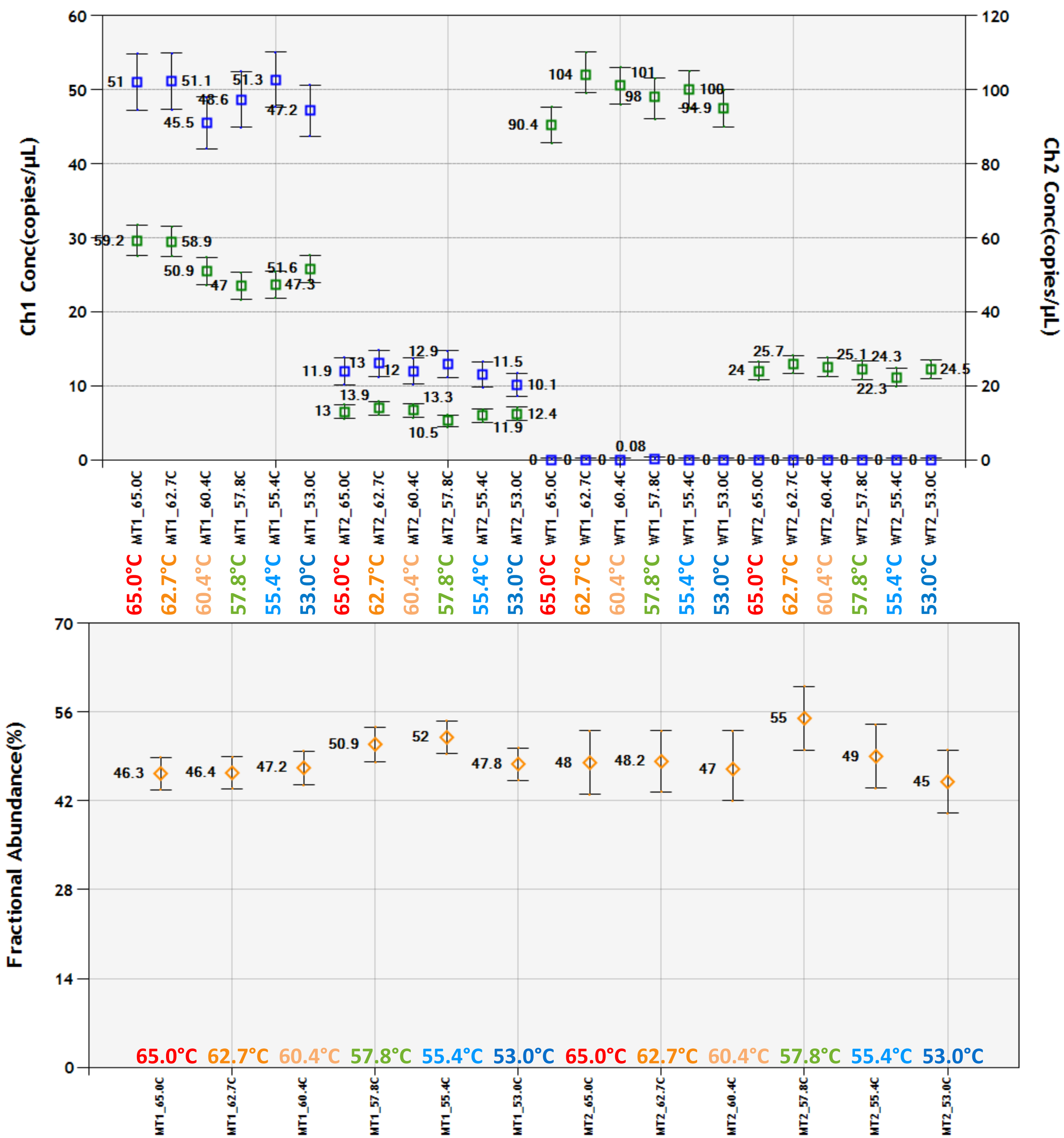

S:\Science & Technology\UK\Teddington\Programmes\CBM-2\Projects\Horizon TSB\WP3\Data\Exp051 NRAS A59T & EGFRins assay optimisation (QX200)

NRAS A59T FAM/Cal Fluo Orange 650 (FAM/HEX settings). Same threshold settled for each temperature

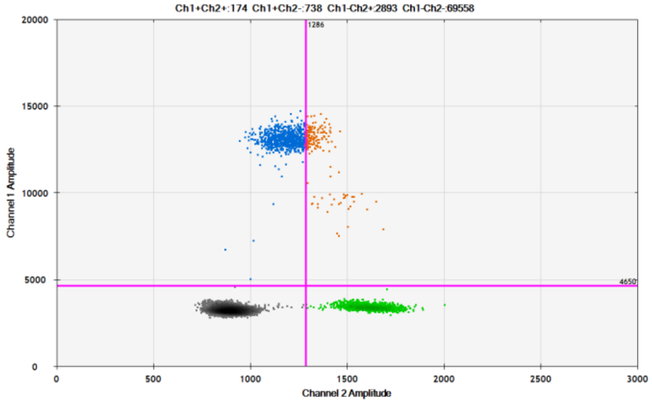

MT λ1 62.7 °C

(intact; ≈ 8 ng/rxn)

"lasso corrected"

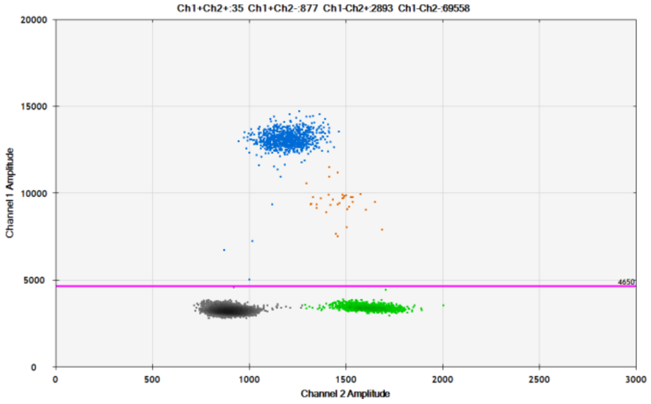

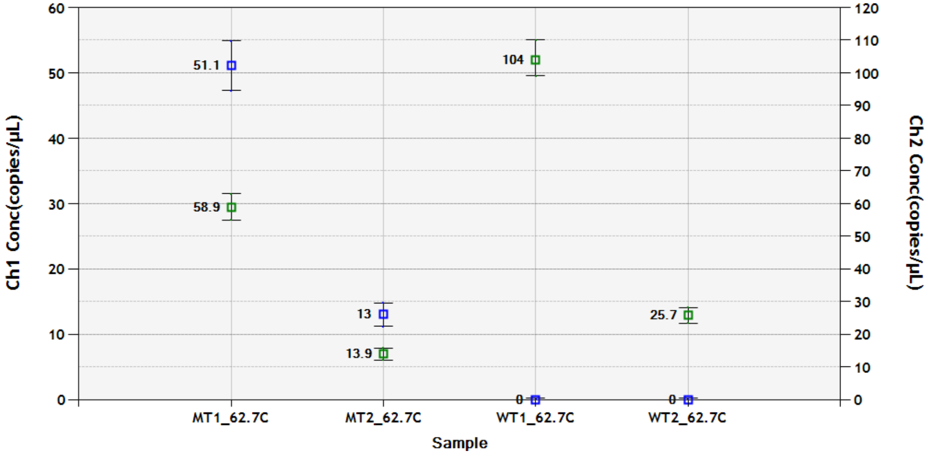

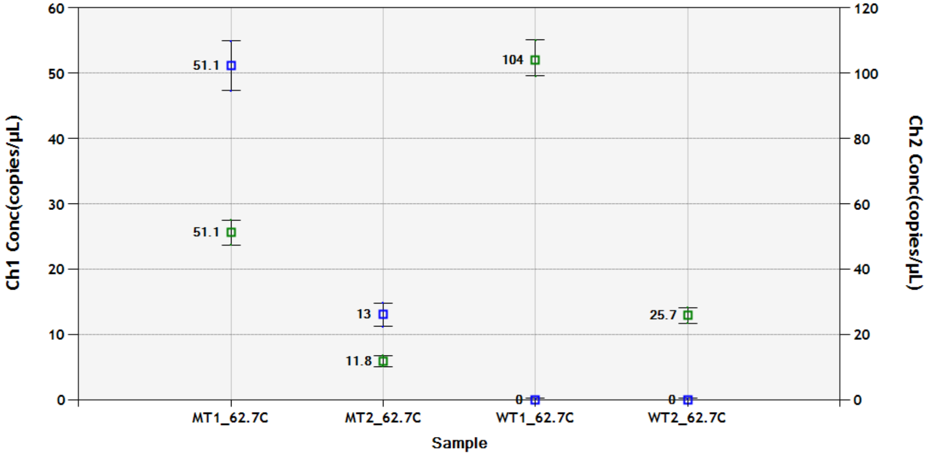

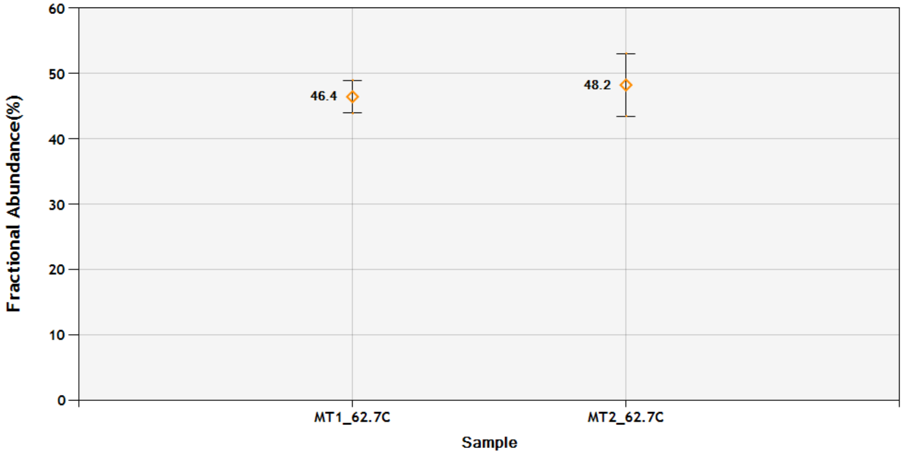

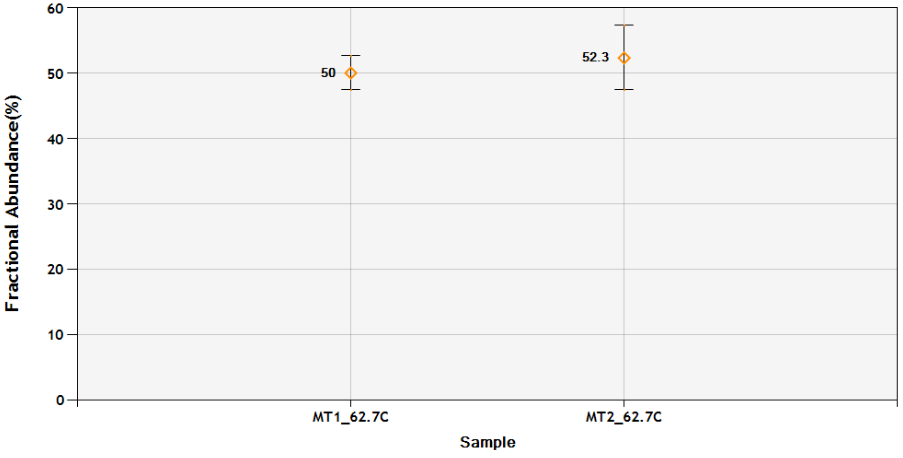

Assay: PI3KCA E545K/WT (FAM/Cal Fluo Orange 650 (FAM/HEX settings)).

MT λ1 65.0 °C (intact; ≈ 40 ng/rxn)

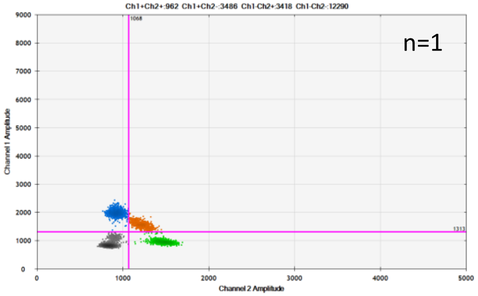

MT λ1 65.0 °C (intact; ≈ 8 ng/rxn)

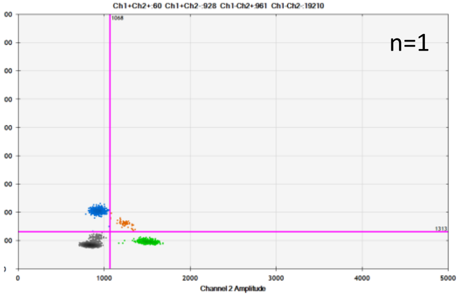

WT λ1 65.0 °C (intact; ≈ 40 ng/rxn)

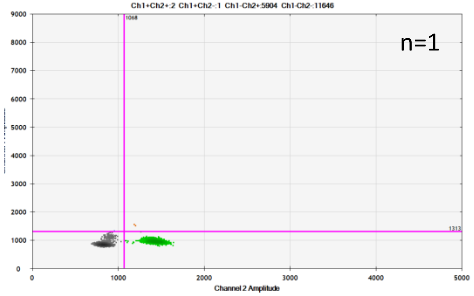

WT λ1 65.0 °C (intact; ≈ 8 ng/rxn)

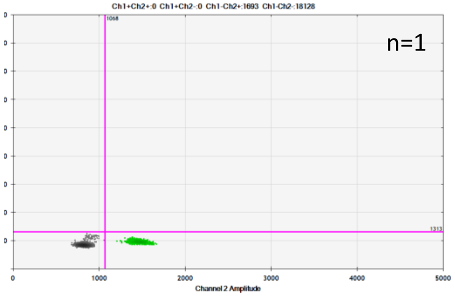

Threshold=> Ch1: 1313, Ch2: 1068

MT λ1 62.7 °C (intact; ≈ 40 ng/rxn)

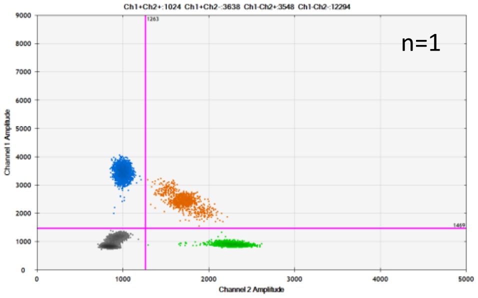

MT λ1 62.7 °C (intact; ≈ 8 ng/rxn)

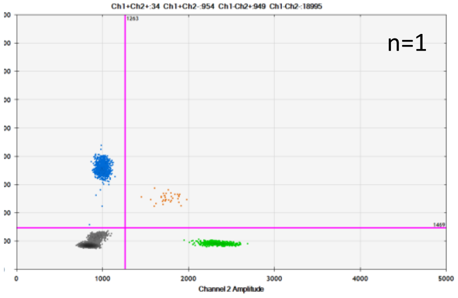

WT λ1 62.7 °C (intact; ≈ 40 ng/rxn)

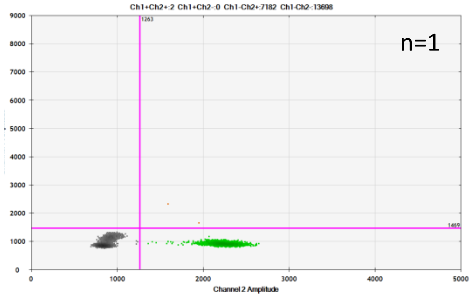

WT λ1 62.7 °C (intact; ≈ 8 ng/rxn)

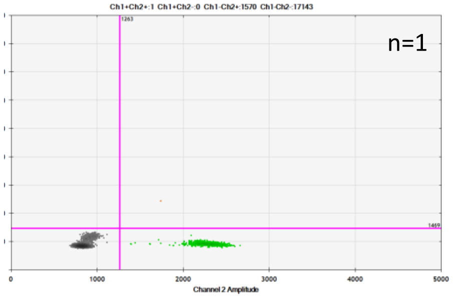

Threshold=> Ch1: 1469, Ch2: 1263

MT λ1 60.4 °C (intact; ≈ 40 ng/rxn)

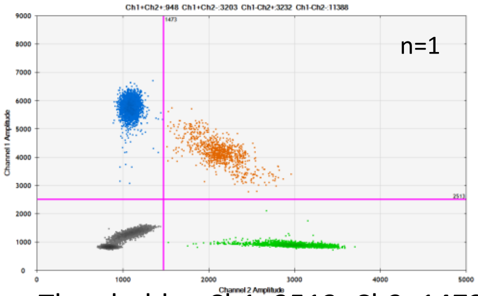

MT λ1 60.4 °C (intact; ≈ 8 ng/rxn)

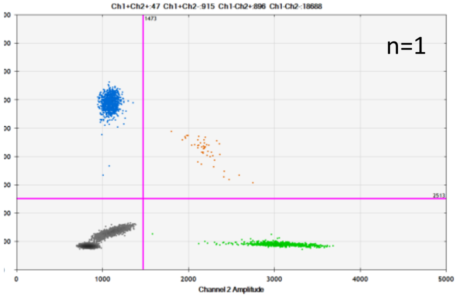

WT λ1 60.4 °C (intact; ≈ 40 ng/rxn)

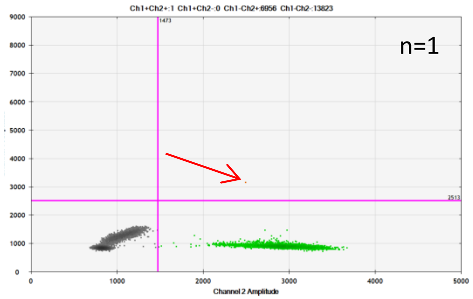

WT λ1 60.4 °C (intact; ≈ 8 ng/rxn)

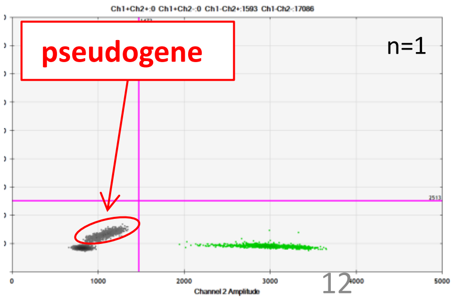

Threshold=> Ch1: 2513, Ch2: 1473

PI3K E545K FAM/Cal Fluo Orange 650 (FAM/HEX settings). Lasso was used to define the different populations of droplets (all wells within the same temperature at once)

MT λ1 57.8 °C
(intact; ≈ 40 ng/rxn)

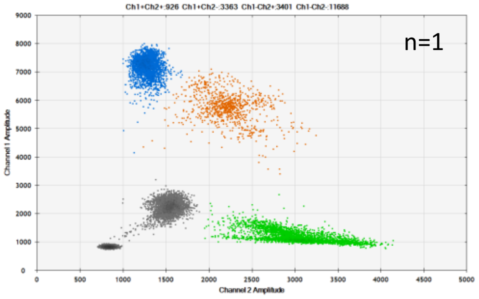

MT λ1 57.8 °C
(intact; ≈ 8 ng/rxn)

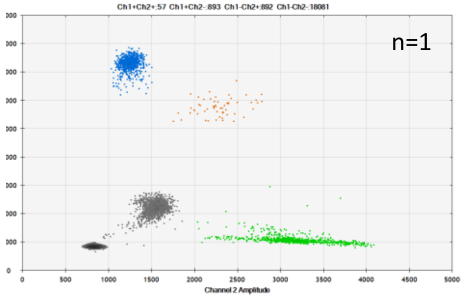

WT λ1 57.8 °C
(intact; ≈ 40 ng/rxn)

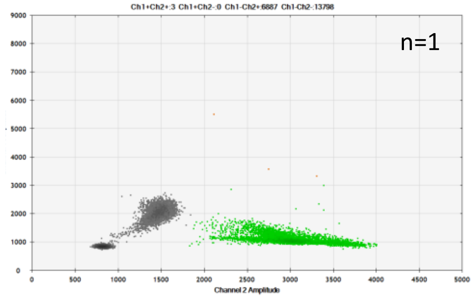

WT λ1 57.8 °C
(intact; ≈ 8 ng/rxn)

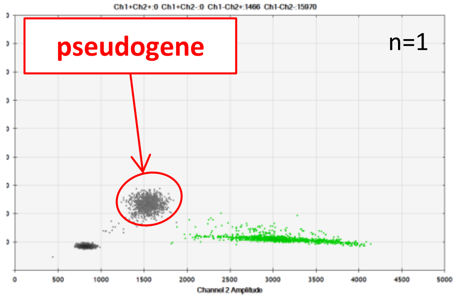

MT λ1 55.4 °C
(intact; ≈ 40 ng/rxn)

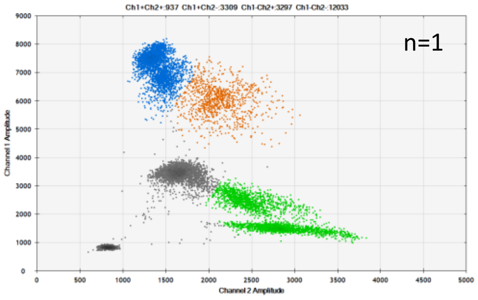

MT λ1 55.4 °C
(intact; ≈ 8 ng/rxn)

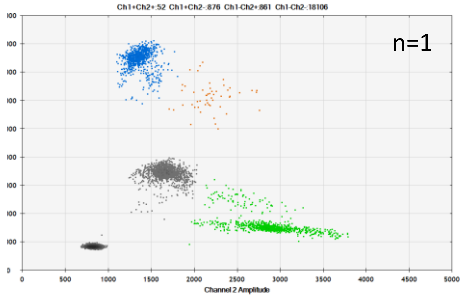

WT λ1 55.4 °C
(intact; ≈ 40 ng/rxn)

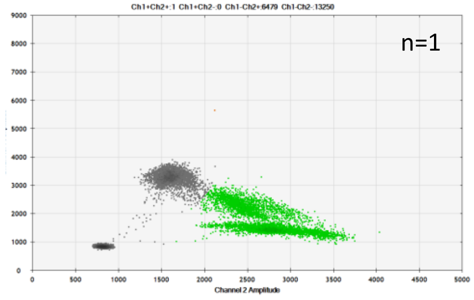

WT λ1 55.4 °C
(intact; ≈ 8 ng/rxn)

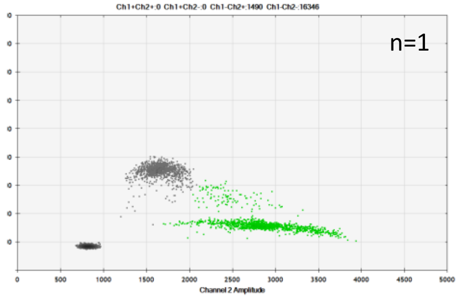

MT λ1 53.0 °C
(intact; ≈ 40 ng/rxn)

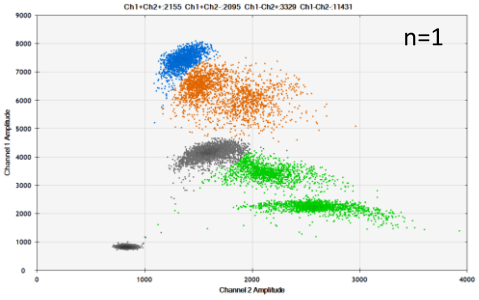

MT λ1 53.0 °C
(intact; ≈ 8 ng/rxn)

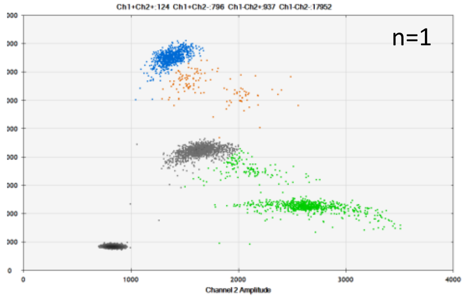

WT λ1 53.0 °C
(intact; ≈ 40 ng/rxn)

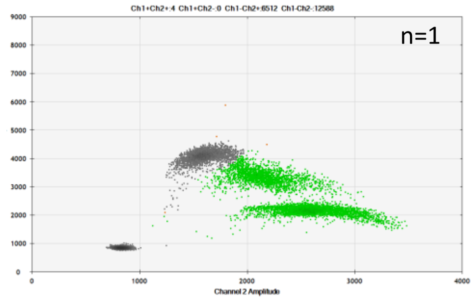

WT λ1 53.0 °C
(intact; ≈ 8 ng/rxn)

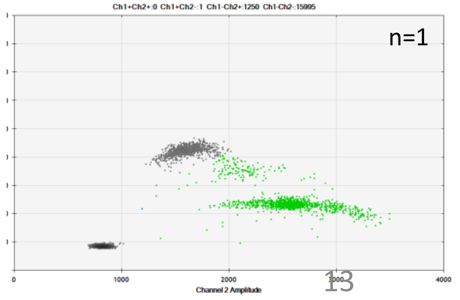

PI3K E545K FAM/Cal Fluo Orange 650 (FAM/HEX settings). Lasso was used to define the different populations of droplets (all wells within the same temperature at once)

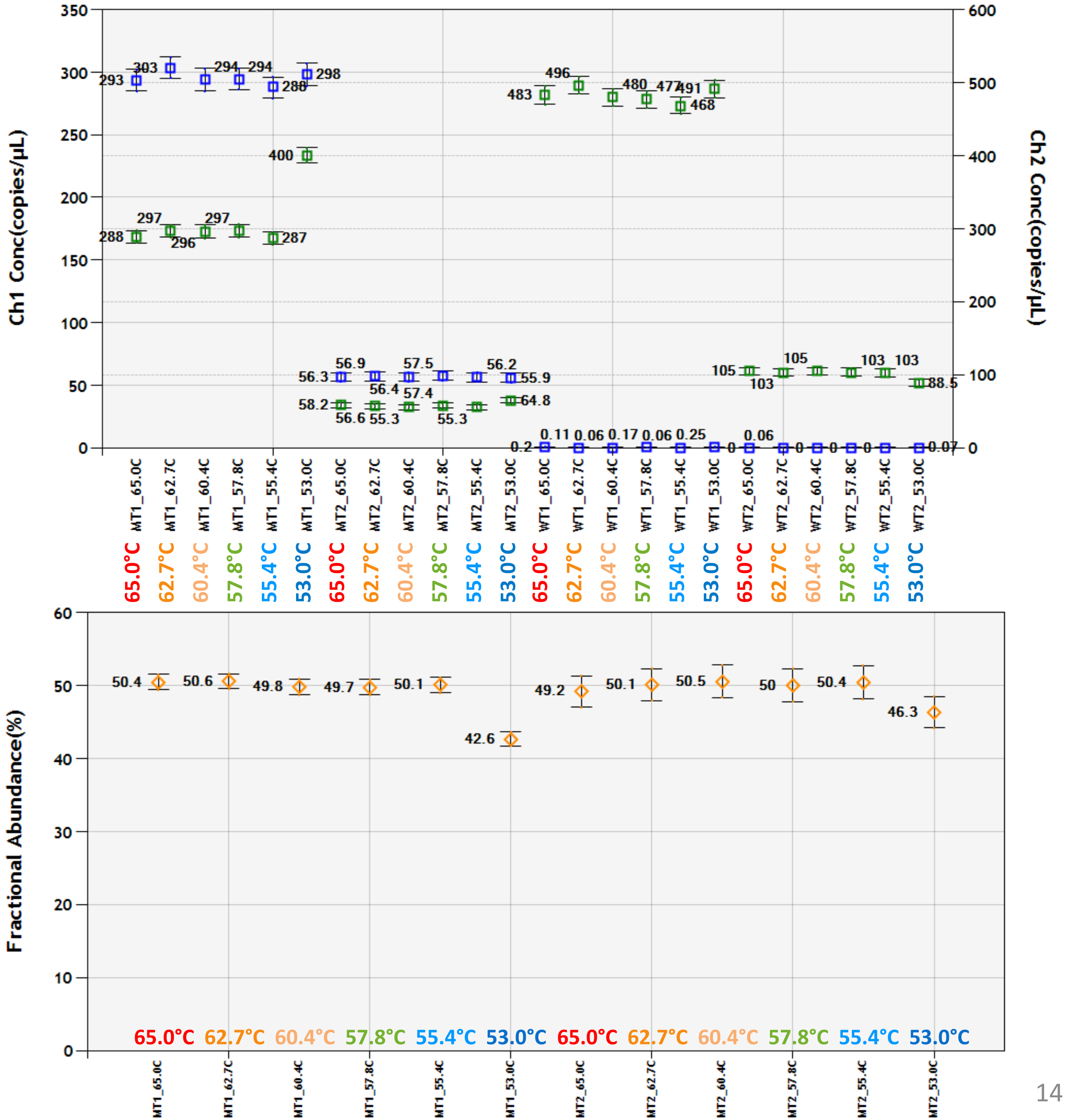

3. dPCR specificity testing (wet lab)

- Assays were tested for cross-reactivity with mutant targets in the same gene or homologous genes within the Multiplex I cfDNA Reference Standard panel using Horizon Reference Standards with 50% mAF of the non-specific mutant (40 ng/reaction) (n = 3).
- Both the presence of false positives (mutant probe binding non-specific template) or false negatives (wild-type probe not binding non-specific mutant template) were investigated
- No significant increases in mutant FPR or concentration of wild-type DNA were measured

Assay	Test template for specificity testing (Horizon P/N)		
EGFR L858R	EGFR T790M (HD-C886)	EGFRΔE746-A750 (HD-C887)	V769_D770insASV (HD-C888)
KRAS G12D	NRAS A59T (HD-C891)	NRAS Q61K (HD-C890)	
NRAS A59T	KRAS G12D (HD-C889)	NRAS Q61K (HD-C890)	
PI3KCA E545K	N/A No other mutations to PIK3CA present in panel		

Specificity testing (1): EGFR L858R/WT

EGFR L858R assay (BR)
EGFR L858R template
(50% MT; 40ng/rxn)

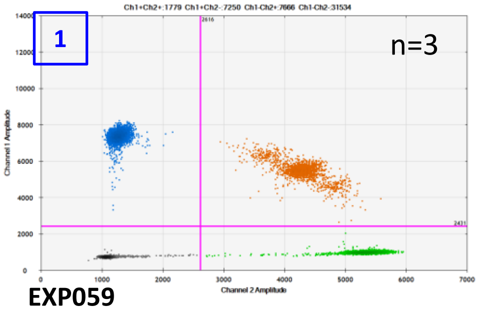

EXP059

EGFR L858R assay (BR)
EGFRdel template
(50% MT; 40ng/rxn)

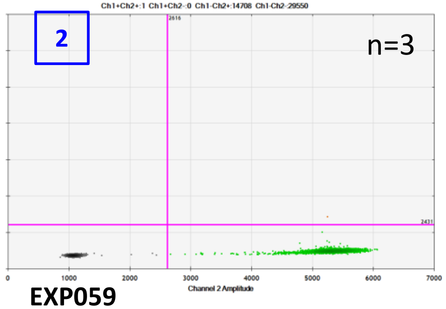

EXP059

EGFR L858R assay (BR)
EGFRins template
(50% MT; 40ng/rxn)

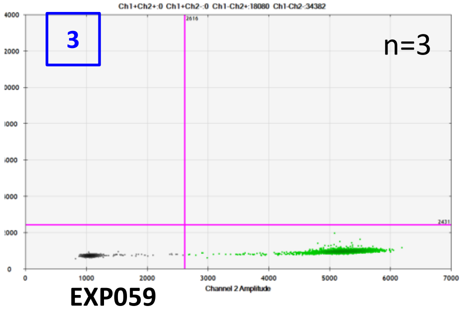

EXP059

EGFR L858R assay (BR)
EGFR T790M template
(50% MT; 40ng/rxn)

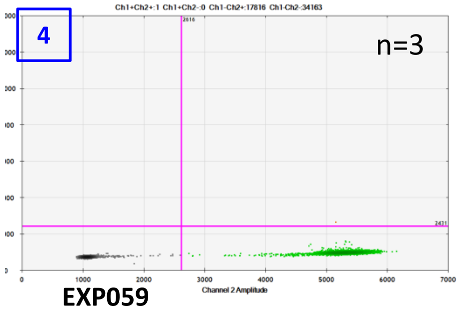

EXP059

MT FPR (100% WT DNA): 0.001%

MT FPR ~ 0.006%

MT FPR ~ 0.000%

MT FPR~ 0.004%

WT detection with EGFR L858R assay (BS)

		**Average (c/µL)**	Fold change
Specific	**MT1 L858R 1**	**1026**	
	MT1 x 2 (≈WT1)*	**2051**	
Non-specific	MT1 del 2	**1906**	0.93
	MT1 ins 3	**1988**	0.97
	MT1 T790M 4	**1975**	0.96

*We didn't have WT1 template in this experiment, so we worked out the expected c/µL as the double of the one obtained in the MT1 template (50% mAF)

Specificity testing (2): KRAS G12D/WT

KRAS G12D assay (BR)
KRAS G12D template
(50% MT; 40ng/rxn)

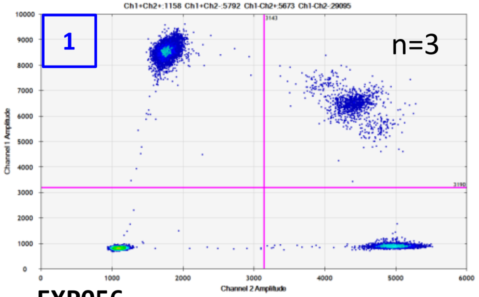

EXP056

KRAS G12D assay (BR)
KRAS WT template
(100% WT; 40ng/rxn)

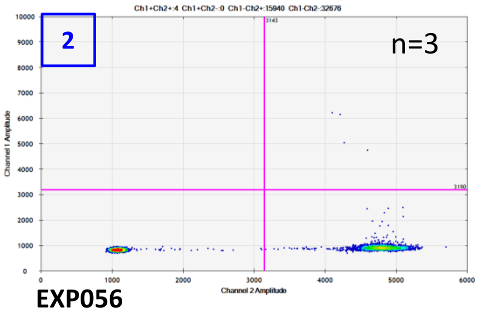

EXP056

MT FPR ≈ 0.020%

KRAS G12D assay (BR)
NRAS Q61K template
(50% MT; 40ng/rxn)

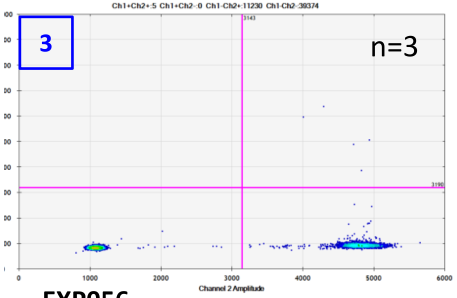

EXP056

MT FPR ~ 0.030%

KRAS G12D assay (BR)
NRAS A59T template
(50% MT; 40ng/rxn)

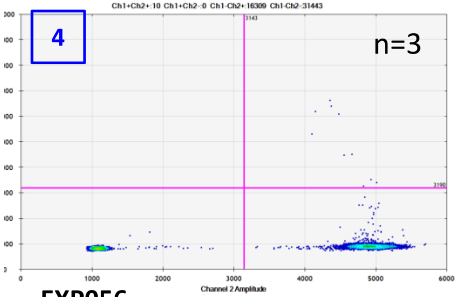

EXP056

MT FPR~ 0.049%

WT detection with KRAS G12D assay (BR)

		**Average (c/µL)**	Fold change
Specific	**G12D MT1 1**	**843**	
	**G12D WT1 2**	**1869**	
Non-specific	A59T MT1 **3**	**1967**	1.05
	Q61K MT1 **4**	**1869**	1.00

EXP056

Specificity testing (3): NRAS A59T/WT

NRAS A59T assay (BS)
NRAS A59T template
(50% MT; 40ng/rxn

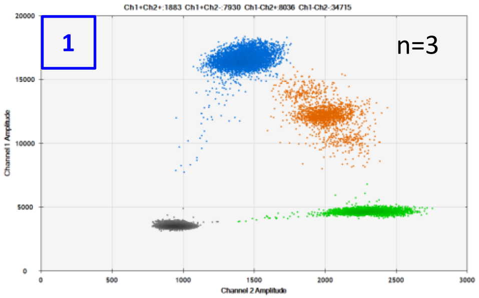

EXP057

NRAS A59T assay (BS)
NRAS WT template
(**100% WT**; 40ng/rxn

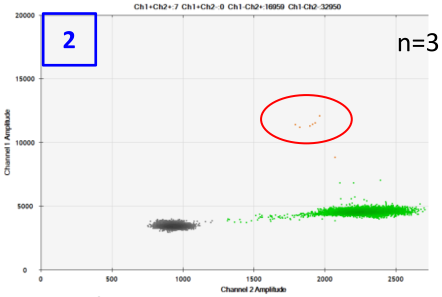

EXP057

MT FPR ≈ 0.035%

NRAS A59T assay (BS)
NRAS Q61K template
(50% MT; 40ng/rxn

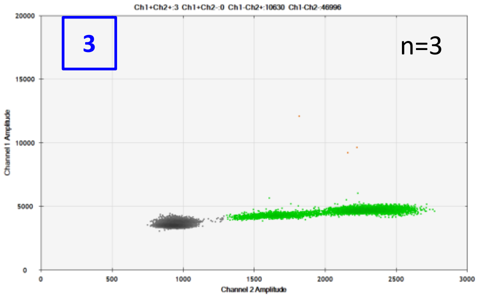

EXP057

MT FPR ~ 0.006%

NRAS A59T assay (BS)
KRAS G12D template
(50% MT; 40ng/rxn

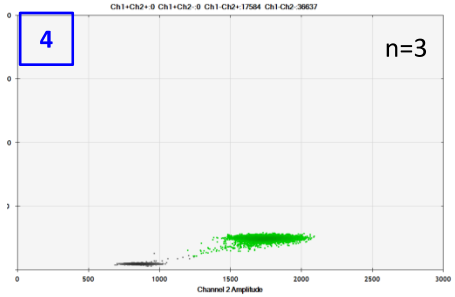

EXP057

MT FPR~ 0.016%

WT detection with NRAS A59T assay (BS)

		**Average (c/µL)**	Fold change
Specific	**MT1** 1	**984**	
	**WT1** 2	**1955**	
Non-specific	NRAS Q61K MT1 3	**1953**	1.00
	KRAS G12D MT1 4	**2117**	1.08
