# Supplementary material for: Development of a highly sensitive liquid biopsy platform to detect clinically-relevant cancer mutations at low allele fractions in cell-free DNA
Source: PLoS One. 2018 Mar 16;13(3):e0194630. doi: 10.1371/journal.pone.0194630 (PMC5856404; doi:10.1371/journal.pone.0194630)
Supplement: S2 Table — A-C. A. dPCR assay information B. dPCR assay information (custom design) C. PCR cycling conditions. (DOCX) [file pone.0194630.s009.docx]

**S2 Table**

**A. dPCR assay information (PrimePCR)**

| Target | MT PrimePCR assay ID (fluorophore) | WT PrimePCR assay ID (fluorophore) | Amplicon size (bp) |
| --- | --- | --- | --- |
| *EGFR* L858R/WT | dHsaCP2000021 (FAM) | dHsaCP2000022 (HEX) | 73 |
| *KRAS* G12D/WT | dHsaCP2500596 (FAM) | dHsaCP2500597 (HEX) | 57 |

**B. dPCR assay information (Custom design)**

| Target | Forward primer sequence (5’-3’) | Reverse primer sequence (5’-3’) | MT probe sequence (5’-3’) | WT primer sequence (5’-3’) | Amplicon size (bp) | Final primer (probe) concentration |
| --- | --- | --- | --- | --- | --- | --- |
| *NRAS* A59T/WT | GATGGTGAAACCTGTTTGTTGGA | TCGCCTGTCCTCATGTATTGG | TACTGGATACAACTGGACAA  (FAM-BHQplus) | CTGGATACAGCTGGACAA  (Cal Fluo Orange 560-BHQplus) | 89 | 0.9 μM  (0.25 μM) |
| *PI3KCA* E545K/WT | GAACAGCTCAAAGCAATTTCTACAC | AGCACTTACCTGTGACTCCATAG | TCTGAAATCACTAAGCAGGA  (FAM-BHQplus) | CTGAAATCACTGAGCAGGA  (Cal Fluo Orange 560-BHQplus) | 89 | 0.9 μM  (0.25 μM) |

**C. PCR cycling conditions**

| Step | Time | Temp (°C) | Ramp rate (°C) | # of cycles |
| --- | --- | --- | --- | --- |
| Enzyme activation | 10 min | 95 | 2 | 1 |
| Denaturation | 30 sec | 94 | 2 | 40 |
| Annealing/extension | 1 min | 55.0* / 60.4** | 2 |  |
| Enzyme Heat Kill | 10 min | 98 | 2 | 1 |
| Hold (optional) | forever | 12 | 1 | 1 |

*PrimePCR assays **Custom assay
