# Supplementary material for: Development of a highly sensitive liquid biopsy platform to detect clinically-relevant cancer mutations at low allele fractions in cell-free DNA
Source: PLoS One. 2018 Mar 16;13(3):e0194630. doi: 10.1371/journal.pone.0194630 (PMC5856404; doi:10.1371/journal.pone.0194630)
Supplement: S3 Table — (DOCX) [file pone.0194630.s010.docx]

**S3 Table. LOD of dPCR assays**

| Target | dPCR assay LOD (% mAF) |
| --- | --- |
| *EGFR* L858R | 0.041% |
| *KRAS* G12D | 0.076% |
| *NRAS* A59T | 0.097% |
| *PI3KCA* E545K | 0.124% |
